# Memory CD8^+^ T cell heterogeneity is primarily driven by pathogen-specific cues and additionally shaped by the tissue environment

**DOI:** 10.1016/j.isci.2020.101954

**Published:** 2020-12-16

**Authors:** Esmé T.I. van der Gracht, Guillaume Beyrend, Tamim Abdelaal, Iris N. Pardieck, Thomas H. Wesselink, Floortje J. van Haften, Suzanne van Duikeren, Frits Koning, Ramon Arens

**Affiliations:** 1 Department of Immunology, Leiden University Medical Center, Leiden 2333ZA, the Netherlands; 2 Delft Bioinformatics Lab, Delft University of Technology, Delft 2628XE, the Netherlands; 3 Leiden Computational Biology Center, Leiden University Medical Center, Leiden 2333ZA, the Netherlands

**Keywords:** Immunology, Cell Biology

## Abstract

Factors that govern the complex formation of memory T cells are not completely understood. A better understanding of the development of memory T cell heterogeneity is however required to enhance vaccination and immunotherapy approaches. Here we examined the impact of pathogen- and tissue-specific cues on memory CD8^+^ T cell heterogeneity using high-dimensional single-cell mass cytometry and a tailored bioinformatics pipeline. We identified distinct populations of pathogen-specific CD8^+^ T cells that uniquely connected to a specific pathogen or associated to multiple types of acute and persistent infections. In addition, the tissue environment shaped the memory CD8^+^ T cell heterogeneity, albeit to a lesser extent than infection. The programming of memory CD8^+^ T cell differentiation during acute infection is eventually superseded by persistent infection. Thus, the plethora of distinct memory CD8^+^ T cell subsets that arise upon infection is dominantly sculpted by the pathogen-specific cues and further shaped by the tissue environment.

## Highlights

• Heterogeneous subsets of both circulating and tissue-resident memory CD8^+^ T cells exist
• Memory CD8^+^ T cell heterogeneity is profoundly sculpted by pathogen-specific cues
• Memory CD8^+^ T cell heterogeneity is additionally shaped by the tissue environment
• Viral persistance supersedes memory CD8^+^ T cell differentiation after acute infection

## Graphical Abstract

**Figure:**
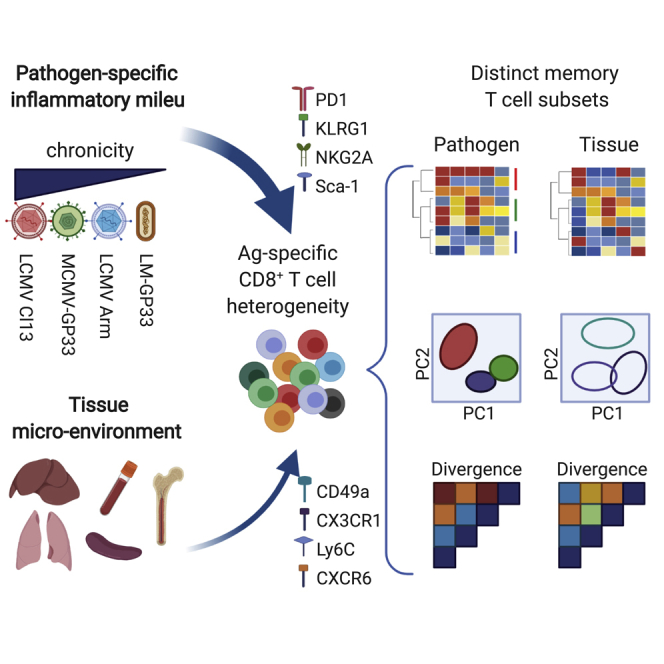

## Introduction

After infection, naive antigen-specific CD4^+^ and CD8^+^ T cells clonally expand and differentiate into effector cell populations, which further segregate into phenotypically diverse long-lived memory T cell subsets that provide protection upon (re-)infection (Arens and Schoenberger, 2010). These memory T cell populations reside in the blood circulation and lymphoid organs, but also in non-lymphoid tissues (Casey et al., 2012; Gebhardt et al., 2009; Masopust et al., 2010; Schenkel et al., 2014). Memory T cells are classically divided into two major subsets based on their circulatory patterns: central-memory T (T_CM_) cells patrol secondary lymphoid organs, governed by expression of homing molecules CD62L and CCR7, whereas effector-memory T (T_EM_) cells lack these homing molecules, which enables them to recirculate through non-lymphoid organs (Sallusto et al., 2004). More recently, a third major subset of memory T cells has been identified based on their restricted recirculation capacity. These tissue-resident memory T (T_RM_) cells are characterized by the expression of CD69 and are found in virtually every tissue (Rosato et al., 2017).

The pivotal role of T cells as mediators and sentinels of immune homeostasis during health and disease drove the necessity of comprehensive assessment of memory T cell heterogeneity. The identification of subpopulations based on phenotypic changes has indeed been very useful to understand memory T cell development and to perceive changes in immune homeostasis during disease. For example, distinct types of T cell subsets are linked to infectious disease, cancer, and autoimmune disease (Ahlers and Belyakov, 2010; van der Leun et al., 2020). This increased understanding of heterogeneity within the memory T cell pool coincided with the advances in cytometry and the availability of monoclonal antibodies. Moreover, cytometry can also be combined with MHC tetramer technology, which provides insights into the characteristics of antigen-specific T cells within the total T cell pool (Davis et al., 2011).

The multiparametric breadth of mass cytometry and the high-dimensional analysis of the data provide the ideal setting to deeply comprehend the complex diversity of T cell subsets and T cell states (Cheng and Newell, 2016; Spitzer and Nolan, 2016). By incorporating markers that identify various cellular properties (i.e., differentiation, function, and/or trafficking) into the T cell panel, one could evaluate the heterogeneous profiles of memory T cells in different virus infections concurrently and unbiased to resolve outstanding questions surrounding the impact of, e.g., organ-specific imprinting versus pathogen-specific inflammation on memory cell differentiation. Previously, the importance of pathogen-specific cues on memory T cell differentiation has been studied, showing that different types of infections lead to particular differentiation types (Appay et al., 2002; Obar et al., 2011; Plumlee et al., 2013). However, these earlier studies lack a deep dissection of memory CD8^+^ T cell heterogeneity. In addition, it remained unaddressed whether there is a hierarchical contribution of infection or tissue-specific cues to memory T cell differentiation.

Here we examined the impact of pathogen- and tissue-specific cues on the development of the heterogeneous populations of memory CD8^+^ T cells simultaneously. We assessed the CD8^+^ T cell heterogeneity using high-dimensional mass cytometry with 41 markers including indicators for recognizing antigen-specific T cells, and we interrogated the impact of tissue-specific imprinting in synchronized yet diverse pathogen-modulated milieus. Distinct pathogen-specific T_CM_, T_EM_, and T_RM_ cell subsets were recognized, and albeit to a lesser extent, also tissue-specific signals shaped the differentiation of memory CD8^+^ T cells. Thus, memory T cell heterogeneity is strongly shaped by the pathogen-specific inflammatory milieu and in addition shaped by the tissue micro-environment.

## Results

### Pathogen-specific cues during acute infection shape the development of distinct CD8^+^ T cell differentiation subsets

**Figure 1:**
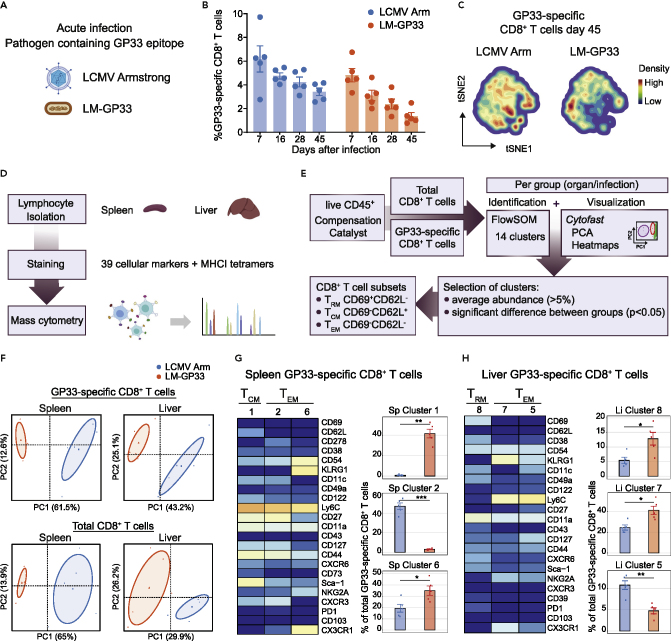
Pathogen-specific cues during acute infection shape the development of distinct CD8^+^ T cell subsets (A) C57BL/6 mice were infected with LCMV Armstrong or LM-GP33. (B) Longitudinal analysis of GP33-specific CD8^+^ T cells in blood. Data are represented as mean ± SEM. Dots represent the values from individual mice. (C) tSNE maps describing the local probability density of GP33-specific CD8^+^ T cells stained with CD62L, CD44, and KLRG1 at day 45 post-infection. (D) Schematic of the mass cytometric analysis of lymphocytes isolated from spleen and liver. (E) Mass cytometry data analysis workflow. (F) Principal Component Analysis (PCA) of mass cytometry data illustrating the phenotypic dissimilarity of GP33-specific and total CD8^+^ T cell clusters in spleen and liver induced by disparate infections (day 50 post-infection). (G and H) Heatmaps of splenic (G) and liver (H) GP33-specific CD8^+^ T cell clusters. Clusters were selected on their abundance (>5%) and significant difference and categorized into T_CM_, T_EM_, and T_RM_ cell subsets. The level of ArcSinh5 transformed marker expression of the markers providing discernment is displayed by a rainbow scale. Bar graphs indicate the abundance and significant differences of the selected GP33-specific CD8^+^ T cell clusters elicited by LCMV Armstrong and LM-GP33 infection. Data are represented as mean ± SEM. ∗P < 0.05, Student t test. See also Figures S1–S3 and Table S1.

To study whether pathogen-specific cues impact memory CD8^+^ T cell heterogeneity, mice were infected with two different pathogens eliciting an acute infection. We used lymphocytic choriomeningitis virus (LCMV) strain Armstrong and *Listeria monocytogenes* (LM) containing the GP33 epitope from LCMV (LM-GP33) (Figure 1A), which allowed us to study the same antigen-specific T cell population in different pathogen-modulated settings. Infections with LCMV Armstrong and LM-GP33 elicited similar high frequencies of GP33-specific CD8^+^ T cell populations in the blood, i.e., 5%–6% of the total CD8^+^ T cell population, which peaked around day 8 post-infection followed by contraction and memory formation (Figures 1B and S1A). Flow cytometric analysis of the GP33-specific CD8^+^ T cells showed that the majority of these cells had a similar effector-memory phenotype based on the markers CD44, CD62L, and KLRG1 (Figures S1B and S1C). However, a more detailed analysis of memory CD8^+^ T cell differentiation by Cytosplore (Höllt et al., 2016), which incorporates approximated t-distributed stochastic neighborhood embedding (A-tSNE) algorithms for subset definition, revealed a difference in the heterogeneity of the GP33-specific CD8^+^ T cells in blood at both the acute and memory phase of infection (Figures 1C, S1D, and S1E). Phenotypic differences were also revealed when analyzing the entire CD8^+^ T cell compartment, comprising both the GP33-specific CD8^+^ T cells and other viral-specific subsets, bystander activated CD8^+^ T cells, and naive CD8^+^ T cells (Figure S1F). Thus, by using unsupervised algorithm-based clustering techniques more distinct deviations in the phenotype of both the pathogen-specific and the total memory CD8^+^ T cell pool can be detected.

To gain a deep insight into the memory T cell heterogeneity in both hematopoietic and non-hematopoietic tissues, T cells from the spleen and liver were isolated at day 50 after infection for subsequent analysis by CyTOF mass cytometry (Figure 1D) with 39 cellular markers that allowed the identification of T cell signatures with an unprecedented depth. The panel consisted of lineage markers and markers specific for cell differentiation, activation, trafficking, and function (Table S1 and Figure S2). In addition, anti-PE and anti-APC antibodies coupled to lanthanides were added to the panel for the detection of PE- and APC-labeled MHC class I GP33 tetramer-binding T cells. Upon selection of live single cells, positive for CD45 (Figure S3A), files were compensated using Catalyst (Chevrier et al., 2018), after which CD8^+^ T cells and tetramer-specific CD8^+^ T cells were selected in FlowJo (Figure S3B). Subsequent analysis of total CD8^+^ T cells or GP33-specific CD8^+^ T cells was performed using FlowSOM or Cytosplore and subsequently by *Cytofast* (Beyrend et al., 2018, 2019a) (Figure 1E).

To gain insight into the putative phenotypic differences within the CD8^+^ T cell pool, we first performed Principal Component Analysis (PCA) based on the cluster frequencies of the GP33-specific memory CD8^+^ T cells. The clusters present in liver and spleen were clearly distinct between LCMV Armstrong and LM-infected mice, indicating pathogen-specific clustering of the GP33-specific CD8^+^ T cell populations (Figure 1F). Moreover, PCA of the total CD8^+^ T cell compartment in liver and spleen also revealed pathogen-specific clustering (Figure 1F).

To reveal which clusters most strongly associate with the pathogen-specificity, we performed *Cytofast* analysis, which generates (1) cluster overviews represented by heatmaps displaying all the markers and (2) quantitative bar graphs with statistics. Based on this, we selected clusters based on the size of the cluster (average abundance >5%) and significance (Figures S3C–S3E). After this selection, we categorized the remaining clusters into the three main memory T cell subsets (i.e., T_CM_, T_EM_, and T_RM_) based on their CD69 and CD62L expression. To create comprehensibility regarding the detailed subset characterization, we generated new heatmaps displaying the selected clusters categorized into the T_CM_, T_EM_, and T_RM_ cell subsets and included those markers providing the distinctiveness of the particular cluster. For clarity, we excluded markers that were used to gate the GP33-specific memory T cells (represented by the inclusion markers CD45, CD8a, CD8b, and MHC class I tetramers and the exclusion markers CD19, TCRgd, and CD4) and markers that were not providing any discernment (for example, due to lack of expression on the GP33-specific memory T cells). Within the selected displayed markers, we further focused on describing mainly the markers that provided the highest level of distinction.

We observed that T_CM_ (CD62L^+^ CD69^−^) and T_EM_ (CD62L^−^ CD69^−^) CD8^+^ T cell clusters differ between LCMV Armstrong and LM-GP33 samples from the spleen, whereas both T_EM_ and T_RM_ (CD62L^−^ CD69^+^) CD8^+^ T cell clusters contributed to the clustering patterns in the liver (Figures 1G and 1H). Within the GP33-specific CD8^+^ T cells, a distinct splenic population of T_CM_ cells expressing Sca-1 (Ly6A/E), Ly6C, CD11a (LFA-1 subunit), and CD27^hi^ (Sp cluster 1) and a T_EM_ cell population expressing CD54 (ICAM-1), KLRG1, and CX3CR1 (Sp cluster 6) connected to LM infection, whereas another T_EM_ cell population characterized by higher levels of CXCR3, CD27^hi^, and CD127 (interleukin-7 receptor alpha [IL-7Rα]) (Sp cluster 2) was more abundant upon LCMV Armstrong infection (Figure 1G). These markers may reflect specific adaptation of the T cells responding to either LCMV or LM infection. For example, expression of the chemokine receptor CX3CR1 identifies subsets with unique effector and migratory properties (Bottcher et al., 2015; Gerlach et al., 2016), and CXCR3 mediates the migratory capacities of these T cell subsets into tissues but is also directly involved in the differentiation of CD8^+^ T cells in response to antigen (Christensen et al., 2006; Hu et al., 2011). Expression of the Ly6 family GPI-anchored surface molecules, Sca-1 and Ly6C, may reflect specific cytokine-mediated induction of these molecules upon infection (DeLong et al., 2018).

Analysis of the liver GP33-specific CD8^+^ T cells revealed differences in T_EM_ cell clusters between LM-GP33 and LCMV Armstrong infection (Figure 1H). Liver T_EM_ cells expressing higher levels of Ly6C, CD127, and CD11c (Li cluster 5) were more abundantly present upon LCMV infection, whereas liver T_EM_ cells expressing higher levels of KLRG1, NKG2A, and CXCR3 were highly LM associated (Li cluster 7). KLRG1 and NKG2A are inhibitory NK cell receptors that are variably expressed on effector-memory phenotype T cells and reflect a lower proliferation potential but a higher cytotoxic capacity (McMahon et al., 2002; Thimme et al., 2005). A T_RM_ cell cluster expressing high levels of CD49a, CD38, CXCR6, CD11a, and CD54 was more abundant upon LM infection (Li cluster 8) (Figure 1H). In line with this, the integrin CD49a is directly involved in tissue residency (Cheuk et al.; Reilly et al., 2020), and also CXCR6, CD11a, and the ectoenzyme CD38 are associated with tissue-resident CD8^+^ T cells (McNamara et al., 2017; Stark et al., 2018). Similar analysis of the total CD8^+^ T cell compartment identified comparable T_CM_ and T_EM_ cell clusters in the spleen that associate with either LM or LCMV infection, albeit the differences were less pronounced (data not shown). Thus, despite that the LM-specific CD8^+^ T cell response directed to other epitopes than GP33 epitope is considerably smaller as the LCMV-specific CD8^+^ T cell response because LCMV elicits large responses to a broad array of antigens other than GP33, we noticed that the observations made with GP33-specific CD8^+^ T cells are reflected in the analysis of the total CD8^+^ T cells in both infections. All together, we conclude that circulating CD8^+^ T cell subsets and T_RM_ cell clusters in the liver and spleen connect uniquely to different acute infections, indicating that distinct and long-lasting memory T cell states are formed associating with pathogen-specific cues in lymphoid organs and tissues.

### Pathogen-specific impact on circulating and tissue-resident memory CD8^+^ T cell subsets

**Figure 2:**
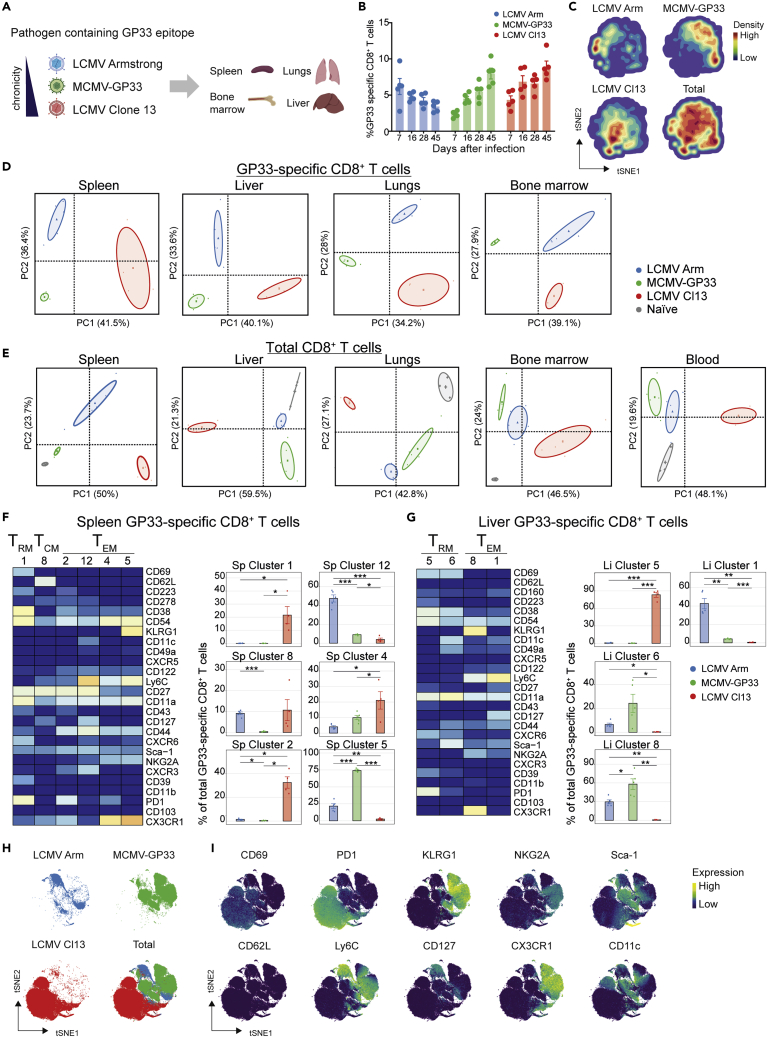
Pathogen-specific impact on circulating and tissue-resident memory CD8^+^ T cell subsets (A) C57BL/6 mice were infected with LCMV Armstrong, MCMV-GP33, or LCMV clone 13. (B) Longitudinal analysis of GP33-specific CD8^+^ T cells in blood. Data are represented as mean ± SEM. Dots represent the values from individual mice. (C) tSNE maps describing the local probability density of GP33-specific CD8^+^ T cells stained with CD62L, CD44, and KLRG1 at day 45 post-infection. (D) Principal Component Analysis (PCA) of mass cytometry data showing the phenotypic dissimilarity of GP33-specific CD8^+^ T cell clusters in spleen, liver, lungs, and bone marrow induced by the different types of infection (day 50 post-infection). (E) PCA of mass cytometry data illustrating the phenotypic dissimilarity of total CD8^+^ T cell clusters in spleen, liver, lungs, bone marrow, and blood of uninfected (naive) mice and of mice infected with different viruses. (F and G) Heatmaps of the splenic (F) and liver (G) GP33-specific CD8^+^ T cell clusters elicited after infection by LCMV Armstrong, MCMV-GP33, or LCMV clone 13. Clusters were selected on their abundance (>5%) and significant difference and categorized into T_CM_, T_EM_, and T_RM_ cell subsets. The level of ArcSinh5 transformed marker expression of the markers provided discernment is displayed by a rainbow scale. Bar graphs indicate the abundance and significant differences of the selected GP33-specific CD8^+^ T cell clusters in each infection. Data are represented as mean ± SEM. ∗P < 0.05, ANOVA. (H and I) tSNE embeddings of liver GP33-specific CD8^+^ T cells isolated from infected mice. (H) Distribution of GP33-specific CD8^+^ T cells per infection in one tSNE analysis. Colors represent the different viral infections. (I) Expression intensity of the cell-surface markers on the GP33-specific CD8^+^ T cells. The color of the cells indicates ArcSinh5-transformed expression values for a given marker analyzed. See also Figures S4 and S5.

To comprehend the impact of pathogen persistence on memory CD8^+^ T cell heterogeneity, we examined memory T cell differentiation during different states of pathogen chronicity. For this, T cells from various organs and responding to pathogens provoking either acute, low-level persistent, or high-level chronic infection were analyzed. We selected LCMV Armstrong (acute), MCMV containing the GP33 epitope from LCMV (low-level persistent), and LCMV clone 13 (high-level chronic), because these pathogens elicit disparate infections and allow us to study the CD8^+^ T cell response to the shared GP33 epitope (Figure 2A). As expected, infections with these pathogens elicited high frequencies of GP33-specific CD8^+^ T cell populations in the blood with the anticipated response kinetics. Acute LCMV Armstrong infection resulted in contracted GP33-specific CD8^+^ T cell responses after the peak (day 8 post-infection), whereas chronic LCMV clone 13 and MCMV-GP33 infection resulted in increasing percentages of GP33-specific CD8^+^ T cells in blood over time (Figure 2B).

Cytosplore analysis of the GP33-specific CD8^+^ T cells using the cell surface markers CD44, KLRG1, and CD62L revealed substantial differences in the phenotype of the GP33-specific CD8^+^ T cells between the three different infections (Figure 2C). Next, we performed high-dimensional mass cytometry to gain deep insight into the details of the developed memory CD8^+^ T cell clusters in various hematopoietic and non-hematopoietic tissues at day 50 post-infection. At the overview level, PCA revealed clear pathogen-specific clustering of the GP33-specific CD8^+^ T cells in liver, lungs, spleen, and bone marrow (BM) (Figure 2D) and such clustering was also revealed by PCA of total CD8^+^ T cells (Figure 2E). Note that PCA of total CD8^+^ T cell clusters from naive mice also separate from those existing in the infected mice in all tissues. To reveal which cell clusters were most strongly associated with the pathogen-specificity, we performed *Cytofast* analysis. In the spleen of LCMV-clone-13-infected mice, a unique T_RM_ cell-like cluster was present, typified by expression of PD-1, CD11a, CD38, CD39, CD54, CXCR6, and CD223 (LAG-3) (Sp cluster 1) (Figure 2F). Thus, besides the T_RM_ cell markers these cells also expressed the inhibitory receptors PD-1 and CD223, and the ectonucleotidase CD39, which are all markers associated with T cell exhaustion (Barber et al., 2006; Blackburn et al., 2009; Gupta et al., 2015) and are known to be upregulated upon LCMV clone 13 infection. Remarkably, a T_CM_ cell subset (Sp cluster 8), characterized by expression of CD27^hi^, CD127, and CD278 (ICOS) was found in the spleen upon infection with LCMV Armstrong and LCMV clone 13 but not MCMV. T_EM_ cell populations expressing PD-1, CD44, and CD11a and either CD27^hi^ (Sp cluster 2) or CX3CR1 (Sp cluster 4) were dominantly present in LCMV clone 13 infection. A T_EM_ cell cluster in the spleen expressing Ly6C, CD27, CXCR3, and CD127 (Sp cluster 12) was more abundant in LCMV Armstrong, whereas a KLRG1^+^CX3CR1^+^ subset was more related to MCMV-GP33 infection.

In the liver, a large LCMV-clone-13-associated GP33-specific CD8^+^ T_RM_ cell population (Li cluster 5) was found, expressing PD-1, CD38, CD54, and CD11a, whereas in MCMV-GP33 and LCMV-Armstrong-infected mice a T_RM_ cell cluster characterized by Sca-1, CD38, CD11c, and CD49a was present (Li cluster 6) (Figure 2G). Also, a T_EM_ cell cluster expressing high levels of Ly6C and CD127 (Li cluster 1) associated to LCMV Armstrong was detected in the liver, whereas liver KLRG1^+^CX3CR1^+^NKG2A^+^ T_EM_ cells (Li cluster 8) associated predominantly to MCMV-GP33 infection. To confirm that marker expression profiles connect uniquely to pathogen-specific clustering, we visualized expression of the markers using tSNE analysis of the liver GP33-specific CD8^+^ T_RM_ cell populations (Figures 2H and 2I). Indeed, although markers such as PD-1 are strongly associated to LCMV clone 13 infection, KLRG1, CX3CR1, and NKG2A expression is mostly connected to MCMV, and Ly6C^hi^ and CD127^hi^ expression is connected to LCMV Armstrong (Figure 2I). The pathogen-specific T cell subsets were also confirmed by flow cytometry based on the markers that provided the best distinction (data not shown).

In the lungs, one T_RM_ and one T_EM_ cell cluster expressing PD-1, CD54, and CD11a was identified associating uniquely to LCMV clone 13 (Lu cluster 1 and 2, Figure S4A). Moreover, one T_EM_ cell cluster (Lu cluster 7) expressing Ly6C and CD127 was uniquely related to LCMV Armstrong, whereas upon MCMV-GP33 infection T_EM_ cells with a KLRG1^+^CX3CR1^+^ phenotype were more abundant. In the BM, an LCMV-clone-13-specific T_RM_ cell-like PD-1^+^CD223^+^CD38^+^CXCR6^+^ subset was present (Figure S4B). BM-residing GP33-specific T_EM_ cells expressing high levels of KLRG1 and CX3CR1 were abundantly present in MCMV-GP33-infected mice and moderately existing after LCMV Armstrong infection (BM cluster 1), whereas a Ly6C^high^CD127^+^CXCR3^+^ T_EM_ cell subset (BM cluster 6) associated uniquely to LCMV Armstrong and a Sca-1^high^CD38^+^CD27^+^T_EM_ cell subset (BM cluster 10) was mainly present upon MCMV-GP33 infection. Similar observations were made by analyzing the total CD8^+^ T cell population, comprising both GP33-specific CD8^+^ T cells as well as other antigen-specific CD8^+^ T cells, bystander-activated CD8^+^ T cells, and naive cells, albeit differences were generally less distinct (Figure S5). Thus, T_EM_, T_CM_, and T_RM_ cell subsets in hematopoietic and non-hematopoietic organs connect uniquely to specific pathogens, indicating that pathogen-specific cues during infection profoundly impact memory T cell heterogeneity.

### The tissue environment shapes the differentiation of viral-specific memory CD8^+^ T cell subsets

**Figure 3:**
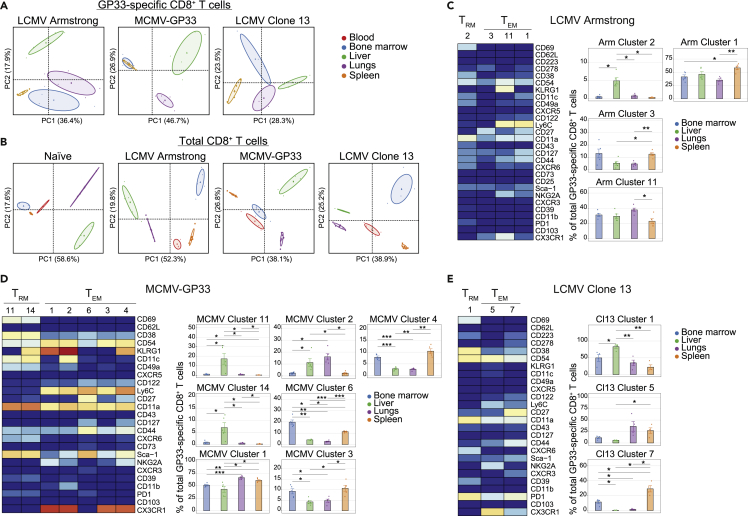
The tissue environment shapes the differentiation of viral-specific memory CD8^+^ T cell subsets (A) Principal Component Analysis (PCA) of mass cytometry data illustrating the phenotypic dissimilarity of GP33-specific CD8^+^ T cell clusters induced by the different tissues (spleen, liver, lungs, and bone marrow) of LCMV-Armstrong-, MCMV-GP33-, or LCMV-clone 13-infected mice (day 50 post-infection). (B) PCA of mass cytometry data illustrating the phenotypic cellular dissimilarity of the total CD8^+^ T cell clusters induced by the different tissues (spleen, liver, lungs, bone marrow, and blood) of uninfected (naive) mice or LCMV-Armstrong-, MCMV-GP33-, or LCMV-clone-13-infected mice. (C–E) Heatmaps of the GP33-specific CD8^+^ T cell clusters elicited after infection with LCMV Armstrong (C), MCMV-GP33 (D), or LCMV clone 13 (E). Clusters were selected on their abundance (>5%) and significant difference and categorized into T_CM_, T_EM_, and T_RM_ cell subsets. The level of ArcSinh5 transformed marker expression of the markers, provided discernment is displayed by a rainbow scale. Bar graphs indicate the abundance and significant differences of the selected GP33-specific CD8^+^ T cell clusters in each tissue. Data are represented as mean ± SEM. ∗P < 0.05, ANOVA. See also Figure S6.

To interrogate whether tissue-specific environmental cues are also able to provide specific imprinting on memory CD8^+^ T cell differentiation, we performed PCA per infection aiming to visualize differences between tissues. PCA of the GP33-specific CD8^+^ T cell clusters separated acute and persistent infection samples (i.e., LCMV Armstrong, MCMV-GP33, LCMV clone 13) from each other based on tissue origin (Figure 3A), except for spleen and BM in MCMV infection where no apparent segregation was seen. PCA based on the cluster frequency values of the total CD8^+^ T cells also showed a separation based on the tissue origin (Figure 3B).

To reveal which cell clusters were most strongly associated with the tissue-specificity, we performed *Cytofast* analysis. Upon LCMV Armstrong infection, a GP33-specific CD11a^+^CD43^+^CD49a^+^CD27^−^ T_RM_ cell subset was specifically located in the liver, and a Sca-1^+^CD122^+^CD27^+^ T_RM_ cell subset was specific for the lungs. Moreover, a CD27^+^Ly6C^−^ T_EM_ cell cluster (Arm cluster 3) was more abundant in BM and spleen, whereas a Ly6C^+^KLRG1^+^ T_EM_ cell cluster (Arm cluster 11) was most abundant in the lungs, and a Ly6C^+^KLRG1^−^ T_EM_ cell cluster (Arm cluster 1) was enriched in the spleen (Figure 3C). Upon MCMV-GP33 infection, two T_RM_ cell clusters expressing CD38, CD11a, CD11c, CXCR6, and Sca-1, typified by divergent KLRG1 expression, connected uniquely to the liver. T_EM_ cells that were NKG2A^+^CX3CR1^+^CD11c^+^ (MCMV cluster 2) were more abundant in the liver and lungs, and three T_EM_ cell subsets (MCMV cluster 3, 4, and 6) typified by high levels of Ly6C, CD11a, and Sca-1 and different in CD27, KLRG1, and CX3CR1 expression were abundant in spleen and BM (Figure 3D). In LCMV clone 13 infection, one T_RM_ cell cluster expressing PD-1, CD38, CD39, CD54, and CXCR6 significantly associated with the liver, whereas a T_EM_ cell subset of cells expressing high levels of PD-1, CX3CR1, NKG2A, and Ly6C (Cl13 cluster 5) was more abundant in the lungs and spleen (Figure 3E). Another PD-1^+^CD27^+^CXCR3^+^ T_EM_ cell cluster (Cl13 cluster 7) connected uniquely to BM and spleen. Similar findings were observed when analyzing the total CD8^+^ T cell compartment (Figure S6). Thus, the tissue environment shapes the heterogeneity of pathogen-specific memory CD8^+^ T cell subsets.

### Memory CD8^+^ T cell heterogeneity is dominantly defined by the type of infection and further shaped by the tissue environment

**Figure 4:**
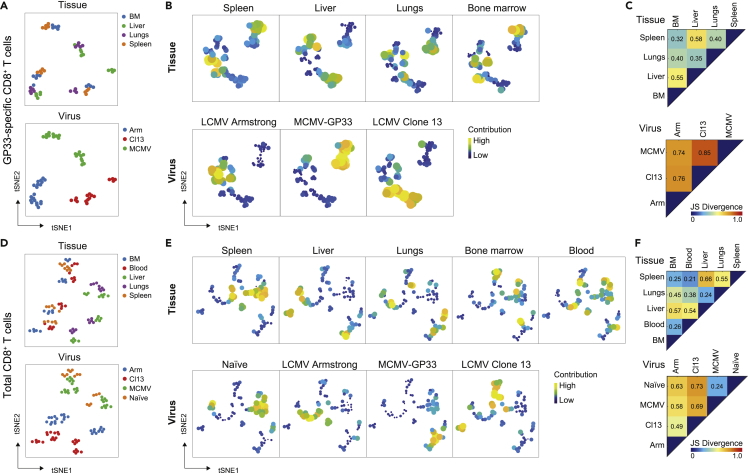
Memory CD8^+^ T cell heterogeneity is dominantly defined by type of infection and further shaped by the tissue environment (A and B) Dual tSNE of memory GP33-specific CD8^+^ T cells (day 50 post-infection) from multiple organs (bone marrow, liver, lungs, spleen) of mice (n = 5 per infection) that received different infections (LCMV Armstrong, LCMV clone 13, MCMV-GP33). (A) tSNE maps showing the 60 samples, color coded per tissue (upper) or per virus (lower). Samples with similar composition across clusters end up close together in the map. (B) tSNE maps showing the 71 GP33-specific CD8^+^ T cell clusters per tissue (upper row) or virus (lower row). Clusters with similar composition profiles across samples end up close together in the map. The varying dot size and color in this cluster tSNE map shows the average cluster normalized frequencies per tissue/virus group, showing the most specific clusters for each tissue/virus group. (C) Pairwise Jensen-Shannon Divergence plots of the tSNE map obtained from all 60 samples of GP33-specific CD8^+^ T cells (see Figures S7A and S7B) grouped by tissue (upper) and virus (lower). (D and E) Dual tSNE analysis of total CD8^+^ T cells from multiple organs (bone marrow, liver, lungs, spleen, blood) of mice that were naive (n = 3) or received different infections (LCMV Armstrong, LCMV clone 13, MCMV-GP33) (n = 5 per infection). (D) tSNE maps showing the 90 samples, color coded per tissue (upper) or per virus (lower). (E) tSNE maps showing the 72 CD8^+^ T cell clusters per tissue (upper row) or virus (lower row). The varying dot size and color in this cluster tSNE map shows the average cluster normalized frequencies per tissue/virus group, showing the most specific clusters for each tissue/virus group. (F) Pairwise Jensen-Shannon Divergence plots of the tSNE map obtained from all 90 samples of total CD8^+^ T cells (see Figures S7C and S7D) grouped by tissue (upper) and virus (lower). See also Figure S7.

To interrogate whether a hierarchy exists between the influence of infection versus tissue-specific cues on memory CD8^+^ T cell differentiation, we performed a system-wide analysis of memory CD8^+^ T cell subsets in all tissues examined after three different types of infections. For this, we integrate collectively the pathogen- and tissue-specific signatures of the memory T cells at day 50 post-infection, which allows to compare the impact of infection type and tissue location. First, we performed a dual tSNE analysis (van Unen et al., 2016) on all 60 samples of GP33-specific CD8^+^ T cells (three types of infection, four tissues, n = 5) based on the abundance of cell clusters, having 71 GP33-specific CD8^+^ T cell clusters. We visualized the segregation of the samples, based on tissue-associated patterns or virus-specific patterns (Figure 4A). The distribution of samples showed clusters of BM and spleen or lungs and liver but also clusters of all four tissues were evident. Clustering based on the type of infection showed strong grouping for each infection. In addition to the sample distribution, visualization of the tSNE map values of the GP33-specific CD8^+^ T cell clusters corroborated the tissue- and virus-associated patterns (Figure 4B). The GP33-specific CD8^+^ T cell clusters contributing to the organ-specific phenotypes had considerable overlap, whereas the distribution of the clusters induced upon LCMV Armstrong, MCMV-GP33, and LCMV clone 13 infection indicated unique virus-specific clustering.

Next, we visualized the similarity between GP33-specific CD8^+^ T cells that were present in spleen, liver, lungs, and BM upon LCMV Armstrong, MCMV-GP33, and LCMV clone 13 infection in one tSNE analysis (Figures S7A and S7B). This analysis corroborated that distinct patterns across different infections exist, whereas tissues provide both overlapping and unique profiles. Subsequently, the similarity between the memory CD8^+^ T cell clusters either in different tissues or in different infections was determined by performing a Jensen-Shannon (JS) divergence analysis. The similarity of a pair of tSNE plots is shown by JS divergence, where a higher JS divergence value indicates more dissimilarity between a pair of tSNE plots. A low JS distance was found when comparing spleen with BM, indicating high similarities between cells residing in these tissues, whereas a higher JS distance was found when comparing spleen with liver and lungs (Figure 4C). In contrast, for all infections a high JS distance was obtained, indicating that these infections induce clear dissimilar virus-specific CD8^+^ T cell states.

In addition to the analysis of the GP33-specific CD8^+^ T cells, we evaluated similarly the total CD8^+^ T cell compartment. Dual tSNE analysis exposed segregation of hematopoietic and non-hematopoietic tissues: spleen samples associated more with BM and blood samples, whereas lungs and liver samples clustered together (Figure 4D). Moreover, infection-associated patterns were also apparent for each infection. The tSNE maps of the total CD8^+^ T cell clusters confirmed the tissue- and virus-associated patterns (Figure 4E). Thus, memory CD8^+^ T cell heterogeneity is strongly influenced by the type of infection, whereas in tissues memory T cell heterogeneity distinction exist between hematopoietic and non-hematopoietic tissues. Still, tissue-specific cues within different hematopoietic and non-hematopoietic tissues shape the phenotype of memory T cells as well. HSNE analysis of the total CD8^+^ T cell compartment corroborated a stronger influence of the type of infection on CD8^+^ T cell phenotype compared with type of tissue (Figures S7C and S7D). However, this was less obvious than the analysis of GP33-specific CD8^+^ T cells because of considerable overlap of CD8^+^ T cells due to the presence of naive subsets present in all infected mice. The JS divergence analysis substantiated high distinctions when comparing different infections, and differences between hematopoietic and non-hematopoietic tissues were also found (Figure 4F). In summary, the integrated systemwide analysis of the T cell compartment revealed that the heterogeneity of CD8^+^ T cell subsets and particularly the antigen-specific memory CD8^+^ T cell populations is strongly defined by the type of infection. Furthermore, diversification of memory T cell formation occurs across hematopoietic and non-hematopoietic tissues.

### Viral persistence is dominantly directing the development of circulating memory CD8^+^ T cell states during co-infection

**Figure 5:**
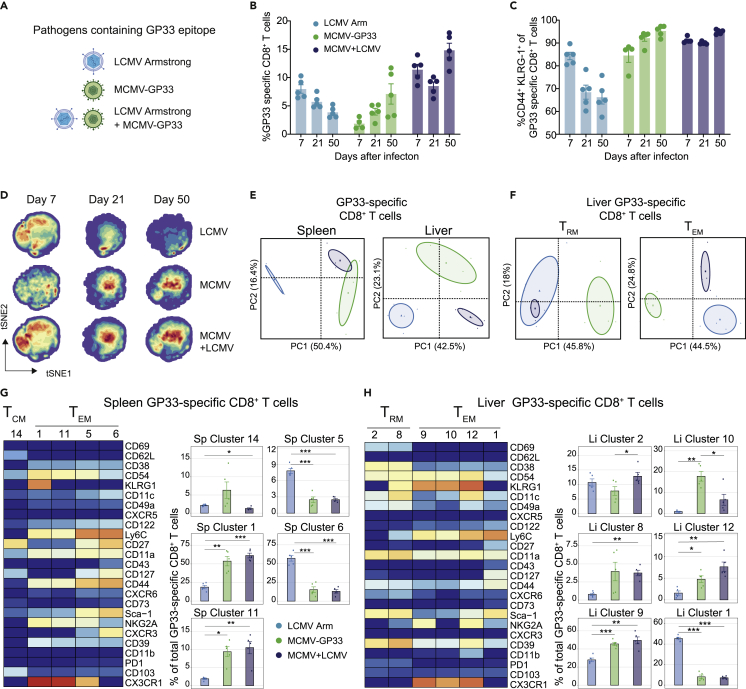
Viral persistence is dominantly directing the development of circulating memory CD8^+^ T cell states during co-infection (A) C57BL/6 mice were infected with LCMV Armstrong, MCMV-GP33, or a combination of LCMV Armstrong and MCMV-GP33 (co-infection). (B and C) Longitudinal analysis of GP33-specific CD8^+^ T cells (B) and CD44^+^KLRG1^+^ GP33-specific CD8^+^ T cells (C) in blood. Data are represented as mean ± SEM. Dots represent the values from individual mice. (D) tSNE maps describing the local probability density of GP33-specific CD8^+^ T cells stained with CD62L, CD44, and KLRG1 at day 7, 21, and 50 post-infection. (E) Principal Component Analysis (PCA) of mass cytometry data illustrating the phenotypic dissimilarity of GP33-specific CD8^+^ T cell clusters in spleen and liver induced by the different types of infection (day 50 post-infection). (F) PCA of mass cytometry data illustrating the phenotypic cellular dissimilarity of GP33-specific CD8^+^ T_RM_ and T_EM_ cells CD8^+^ T cell clusters residing in the liver induced by the different types of infection (day 50 post-infection). (G and H) Heatmaps of the splenic (G) and liver (H) GP33-specific CD8^+^ T cell clusters elicited after infection by LCMV Armstrong, MCMV-GP33 or co-infection. Clusters were selected on their abundance (>5%) and significant difference and categorized into T_CM_, T_EM_, and T_RM_ cell subsets. The level of ArcSinh5 transformed marker expression of the markers, provided discernment is displayed by a rainbow scale. Bar graphs indicate the abundance and significant differences of the selected GP33-specific CD8^+^ T cell clusters in each infection. Data are represented as mean ± SEM. ∗P < 0.05, ANOVA. See also Figure S8.

To examine whether simultaneous infection of two pathogens results in analogous pathogen-specific imprinting or unique imprinting, co-infection studies were performed with LCMV Armstrong and MCMV-GP33, and the differentiation of the GP33-specific CD8^+^ T cells, which are able to respond to both viruses in the same host, was compared with single infection (Figure 5A). Co-infection resulted in higher frequencies of circulating GP33-specific CD8^+^ T cells than the single infections during the peak of acute infection and also at day 50 after infection, demonstrating a combined acute and persistent infection (Figure 5B). Co-infection was also exemplified by a consistently increased effector-memory phenotype (i.e., CD44^+^ KLRG1^+^) of the GP33-specific CD8^+^ T cells in blood (Figure 5C).

Cytosplore analysis based on the markers CD44, CD62L, and KLRG1 revealed that at the peak of the effector response, the phenotype of the GP33-specific CD8^+^ T cells in the blood upon LCMV Armstrong infection resembled the phenotype of the GP33-specific CD8^+^ T cells that develop during co-infection. However, at the memory phase the phenotype of GP33-specific CD8^+^ T cells in the co-infected mice was more similar to those in MCMV-GP33-infected mice (Figure 5D). To examine this further in detail, we performed high-dimensional phenotypic analysis of the GP33-specific memory T cells in the spleen and liver by CyTOF mass cytometry at day 50 after infection. PCA analysis showed that splenic GP33-specific CD8^+^ T cells in co-infected mice resembled those in MCMV-GP33-infected mice, whereas in the liver the GP33-specific CD8^+^ T cells clustered per infection (Figure 5E). This indicates a stronger influence of MCMV infection on the memory T cell differentiation in the spleen, whereas in the liver both infections proportionally contribute to the memory T cell heterogeneity. PCA analysis of the liver GP33-specific T_RM_ cells showed a high overlap between LCMV Armstrong and co-infection, whereas the liver GP33-specific T_EM_ cells segregated (Figure 5F), indicating that especially the T_EM_ cells contributed to the segregation.

*Cytofast* analysis confirmed that the splenic GP33-specific T_EM_ cell clusters were dissimilar between LCMV Armstrong and MCMV-GP33 infection, whereas co-infection generally resembled MCMV infection (Figure 5G). For example, T_EM_ cell subsets expressing high levels of CX3CR1 (Sp cluster 1, 11) were equally present in MCMV-GP33 infected and co-infected mice and more abundant as compared with infection with LCMV Armstrong (see also Figure 2F, cluster 4 and 5). In addition, splenic T_EM_ cell subsets expressing high levels of Ly6C and CD127 (Sp cluster 5 and 6) were enriched upon LCMV infection compared with MCMV infection and co-infection.

In the liver, a T_RM_ cell subset expressing CD38, CD39, and Sca-1 (Li cluster 2) was similar in LCMV-infected and co-infected mice, but this population was lower in MCMV-infected mice (Figure 5H). Vice versa, a T_RM_ cell subset expressing KLRG1 in addition to CD38, CD39, and Sca-1 (Li cluster 8) was increased in MCMV and co-infected mice. Moreover, T_EM_ cell subsets in the liver were either associating with a solitary infection with LCMV Armstrong (Li cluster 1; Ly6C^high^CD27^+^CD127^+^Sca-1^+^) or MCMV-GP33 (Li cluster 10; KLRG1^+^CX3CR1^+^) or were found in both infections but elevated upon co-infection (Li cluster 9; KLRG1^+^CX3CR1^+^NKG2A^+^, Li cluster 12; KLRG1^+^CX3CR1^+^NKG2A^+^CD11c^+^CD39^+^). Thus, the phenotype of circulating antigen-specific memory CD8^+^ T cells upon co-infection is mostly defined by the infection persistence, yet pathogen-specific signals during acute infection still programmed the development of unique and long-lasting CD8^+^ T cell subsets, and this was especially observed for T_RM_ cell subsets.

### The programming of memory CD8^+^ T cell differentiation during acute infection is superseded by viral persistance

**Figure 6:**
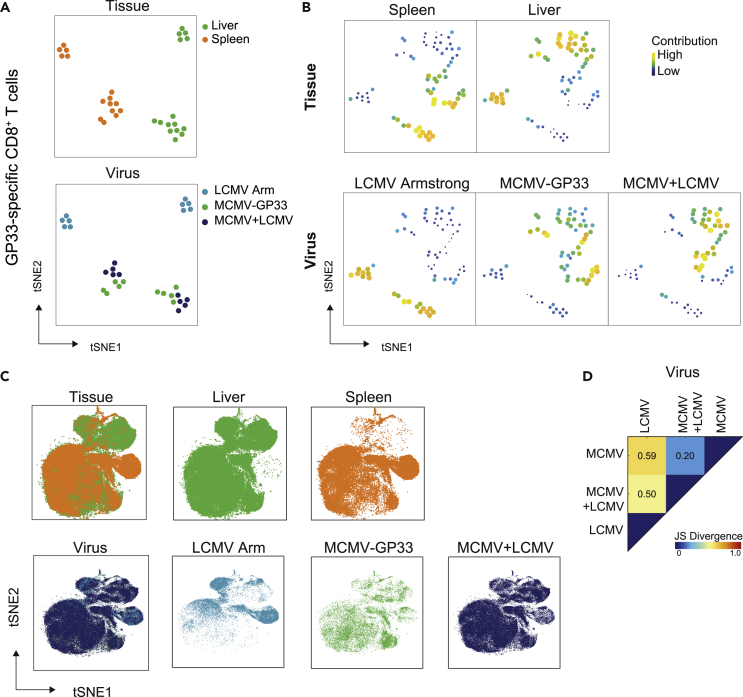
The programming of memory CD8^+^ T cell differentiation during acute infection is superseded by viral persistance (A and B) Dual tSNE analysis of memory GP33-specific CD8^+^ T cells (day 50 post-infection) from liver and spleen of mice (n = 5 per infection) that were infected with LCMV Armstrong, MCMV-GP33, or LCMV Armstrong + MCMV-GP33 (co-infection). (A) tSNE maps showing the 30 samples, color coded per tissue (upper) or per virus (lower). Samples with similar composition across clusters end up close together in the map. (B) tSNE maps showing the 69 GP33-specific CD8^+^ T cell clusters per tissue (upper row) or virus (lower row). Clusters with similar composition profiles across samples end up close together in the map. The varying dot size and color in the cluster tSNE map shows the average cluster-normalized frequencies per tissue/virus group, showing the most specific clusters for each tissue/virus group. (C) GP33-specific CD8^+^ T cells from multiple organs of mice that received different infections analyzed in one tSNE analysis. Cells color coded per type of tissue (upper) or virus (lower). (D) Pairwise Jensen-Shannon Divergence plots of the tSNE maps in (C) grouped by virus.

To analyze the similarity of samples from spleen and liver or the MCMV, LCMV, and co-infection, dual tSNE analysis was performed on all samples of the GP33-specific CD8^+^ T cells present at day 50 post-infection. In total, 69 GP33-specific CD8^+^ T cell clusters were generated by tSNE analysis in Cytosplore. Dual tSNE analysis showed clear distinct tissue-associated patterns, as well as clusters of spleen and liver samples in close proximity of each other (Figure 6A). With respect to virus-specific patterns, MCMV-GP33-associated samples clustered more with co-infection compared with LCMV Armstrong. The tSNE maps of the GP33-specific CD8^+^ T cell clusters validated the large difference in tissue-associated patterns (Figure 6B). This analysis also revealed that GP33-specific CD8^+^ T cell clusters contributing to the MCMV-GP33 and co-infection virus-specific phenotypes segregate apart for LCMV Armstrong, indicating that the majority of the memory T cell phenotypes induced upon MCMV-GP33 are very similar to the co-infection during the memory phase. However, subsets of GP33-specific CD8^+^ T cells associating more to co-infection also exist.

tSNE analysis revealed considerable overlap between liver and spleen but also distinct patterns were apparent. As observed earlier, MCMV infection and LCMV infection induced diverse GP33-specific memory T cell subsets (Figure 6C). Notably, co-infection resembled more the single MCMV infection as compared with single LCMV infection. Nevertheless, also overlays with LCMV infection were existing. Quantification of the similarity by JS divergence analysis verified the low similarities (high JS distance) between cell clusters when MCMV-GP33 was compared with LCMV Armstrong and a high resemblance when comparing MCMV-GP33 with co-infection (Figure 6D).

To corroborate that persistent antigen-triggering has a profound influence on memory T cell differentiation, we infected mice with replicating MCMV or a single-cycle replicating MCMV (MCMV-FKBP), both expressing the model antigen E7 from human papilloma virus (Figure S8), because this allows to directly compare the effect of viral persistence. PCA analysis revealed clear distinction between liver and spleen phenotypes of E7-specific CD8^+^ T cells upon infection with persistent replicating and single-cycle replicating virus (Figure S8), and both T_RM_ and T_EM_ cell subsets contributed to this difference. The CD8^+^ T cell subsets associated with MCMV-E7 infection were characterized with a higher level of KLRG1, CD39, CD11c, and NKG2A expression compared with single-cycle MCMV-FKBP-E7 infection (Figure S8), reflecting a higher activation status due to persistent antigenic triggering.

In summary, we conclude that the phenotypic heterogeneity of antigen-specific CD8^+^ T cells upon co-infection with pathogens eliciting acute and persistent infections is eventually mostly defined by persistent infection, yet pathogen-specific signals during acute infection still program the development of unique and long-lasting CD8^+^ T cell subsets that remain present.

## Discussion

Previous studies have shown that memory T cell heterogeneity exists within the circulating and tissue-resident compartment. However, studies simultaneously comparing the diversity of memory T cell subsets between infections and/or tissues are scarce and mainly restricted to a limited number of cell surface markers. In this study, we performed extensive single-cell phenotypic analysis to interrogate the memory CD8^+^ T cell heterogeneity in various tissues and upon different types of infection simultaneously in mice. Our system-wide analyses revealed substantial heterogeneity in both circulating (central-memory and effector-memory) and tissue-resident CD8^+^ T cell populations. We found that memory CD8^+^ T cell differentiation based on cell-surface markers is foremost defined by the type of infection. Nevertheless, tissue-specific cues within both hematopoietic and non-hematopoietic tissues also uniquely shaped the phenotype of memory CD8^+^ T cells. Correspondingly, the heterogeneity of human T cells in different tissues and upon stimulation has been confirmed by transcriptomic studies, revealing both T cell tissue and activation signatures (Szabo et al., 2019).

Already during clonal expansion, the differentiation patterns of individual CD8^+^ T cells are heterogeneous, as shown by conventional immune profiling and fate mapping (Buchholz et al., 2013; Gerlach et al., 2013). Other studies showed that pathogen- and tissue-specific cues influenced memory T cell differentiation (Badovinac and Harty, 2007; Nolz et al., 2011; Obar et al., 2011; Plumlee et al., 2013); however, this was shown with a limited number of markers, and it remained unanswered whether a hierarchy exist between the influence of the pathogen or tissue on T cell differentiation. Our concurrent evaluation of the high-dimensional phenotypic profiles of CD8^+^ memory T cells in different tissues and upon various virus infections helped to resolve questions and controversies surrounding the particular impact of organ-specific imprinting versus pathogen-specific cues on memory cell differentiation.

In addition to T_EM_ cell subsets and consistent with our data, heterogeneity within T_RM_ cells exists (Kumar et al., 2017, 2018; Milner et al., 2020). The latter is of interest, because these resident CD8^+^ T cells contribute significantly to local immunity (Gebhardt et al., 2009; Masopust et al., 2010; Steinert et al., 2015). More recently, T_RM_ cells have been found to be able to re-enter the circulation upon re-activation, thus subsets of these cells still have recirculation capacity and this contributes to systemic immunity (Behr et al., 2020; Fonseca et al., 2020). We and others observed remarkable distinct T_RM_ cell formation upon chronic LCMV clone 13 infection. Multiple distinct exhausted CD8^+^ T cell subsets were defined, by expression of Ly108 and CD69, with transcriptional, phenotypical, functional, and anatomical differences (Beltra et al., 2020), and by using parabiosed mice it was shown that the resident CD8^+^ T cells in lymphoid tissues after LCMV clone 13 infection have a PD-1^+^ stem-like phenotype (Im et al., 2020).

Besides antigen-dependent signals provided by the TCR, naive CD8^+^ T cells require costimulation and inflammatory cytokines to drive expansion and survival (Arens and Schoenberger, 2010; Curtsinger and Mescher, 2010). We found that persistent antigen-specific triggering eventually dominates over the signals that T cells receive during acute infections with respect to differentiation of circulating memory CD8^+^ T cells. This is likely directly caused by persistent low-level TCR triggering although variances in costimulatory signals may correspondingly influence T cell differentiation (Welten et al., 2013) but also other factors, e.g., differences in local inflammatory cytokines and cellular interactions, can shape the differentiation of CD8^+^ T cells (Enamorado et al., 2018). In lung tissue for example, pulmonary monocytes interact with effector T cells to drive T_RM_ cell differentiation following viral infection (Dunbar et al., 2020). It has recently been shown that lung T_RM_ cells can be reactivated by numerous hematopoietic and non-hematopoietic antigen-presenting cells, but the identity of the antigen-presenting cells influenced the functional properties of the T_RM_ cells (Low et al., 2020). In the liver, Kupffer cells and stromal cells are the sources of soluble mediators (e.g., IL-6, IL-10, and TGFβ) capable of modulating the phenotype of T cells, and the unique signature of such cytokines but also of other factors may further instruct T cells in each tissue type, and this may trigger the phenotypic differences observed. Interestingly, KLRG1^+^CX3CR1^+^ T_EM_ cells, mainly observed upon MCMV infection, were also phenotypically different in various organs. For example, in the lung compartment these T_EM_ cells are co-defined by Ly6C and NKG2A, whereas in BM additionally CD27 and CD122 are expressed.

The apparent PD-1^+^ phenotype in LCMV clone 13 infection likely directly results from strong chronic antigenic triggering, yet tissue-specific signals can still modulate this phenotype, albeit less as compared with milder type of infections. Regarding the liver compartment, our analyses revealed that both heterogeneous T_EM_ and T_RM_ cells accumulate here and contribute to the total memory T cell heterogeneity. In the liver, we found a population of T_RM_ cells expressing CD223 (LAG-3), CXCR6, and CD43 as well as high CD39 and PD-1 levels to represent more than 70% of the total liver antigen-specific CD8^+^ T cells upon chronic LCMV clone 13 infection. In contrast, T_RM_ cell populations induced upon acute LMCV Armstrong or persistent MCMV-GP33 infection displayed a CD11c^+^CD49a^+^Sca-1^+^ phenotype. T_EM_ cell subsets, however, were mainly responsible for the difference between LCMV Armstrong and MCMV-GP33 infections, with Ly6C^+^KLRG1^−^CD127^+^ cells being more abundant in LCMV Armstrong and KLRG1^+^CX3CR1^+^NKG2A^+^ GP33-specific CD8^+^ T_EM_ cells defining the phenotype after MCMV-GP33 infection.

We found large frequencies of pathogen-specific CD8^+^ T cells that display an activated phenotype in tissues that are characterized by high amounts of markers related to inhibitory receptors (PD-1), costimulatory receptors (CD27), cytokine receptors (CD127), NK cell receptors (KLRG1, NKG2A), chemokine receptors (CXCR6, CX3CR1, CXCR3), integrins (CD11c, CD49a), enzymes (CD38), and GPI-anchored membrane proteins (Ly6C, Sca-1). The biological function of these molecules may serve immune checkpoints as has been shown for PD-1, NKG2A, and KLRG1 but may also serve as proteins that provide stimulatory signals as has been shown for CD27 and Ly6C (Bamezai, 2004). Moreover, chemokine receptors and integrins influence migratory properties. How the multitude of all these markers with each having a unique function is translated into functional properties of the memory T cell subsets that cope with the current infection or re-infection is not yet known and will require further investigation.

The distinct phenotypic characteristics of CD8^+^ T_EM_ and T_RM_ cells that differ between diverse types of infection in each tissue represent the enormous plasticity of these cell types. Insight into memory T cell heterogeneity could be used as a resource for the informed design of prognostic and/or therapeutic avenues. In fact, cellular phenotypes can be equally or even more indicative of clinical outcomes than the mere number of infiltrating T cells. For example, the PD-1 pathway blockade increases the numbers of CD4^+^ and CD8^+^ T cell subsets expressing CD278 (ICOS), and co-targeting of this molecule indeed improved efficacy (Beyrend et al., 2019b). The unique imprint of each infection on the memory CD8^+^ T cell differentiation could serve as a valuable tool to study their role in diseases in general and specifically also in tissue-specific immunity. Defining the T cell heterogeneity could accordingly be important to empower immunotherapies targeting specific cell subsets.

Taken together, we show that the plethora of distinct memory CD8^+^ T cell subsets that arise upon infection is sculpted strongly by pathogen-specific signals while tissue-specific imprinting is present but less evident. Our work provides a phenotypic framework for the development of memory CD8^+^ T cells during acute and persistent infection and identifies phenotypically distinct subpopulations in diverse tissues that may play different protective roles in long-term immunity. Further studies examining the plasticity of antigen-specific CD4^+^ T cells could determine whether their heterogeneity is similarly or differentially influenced by infection type and tissue environment. Understanding the pathogen- and tissue-specific memory T cell heterogeneity will have biological implications for designing vaccination regimens against infections. The richness of the T cell heterogeneity regarding cellular states parallels their favorable implications for enhancing vaccination and immunotherapy approaches. In-depth knowledge of the specific T cell signatures across tissues and infections is a major step forward for the rational design of T-cell-targeted immunotherapy strategies.

### Limitations of the study

Here we established that both the infection type and tissue environment play an important role in directing the memory T cell heterogeneity, although a more dominant role for infection was apparent. We used established experimental infection models and performed high-dimensional mass cytometry to effectively analyze the heterogeneity of memory CD8^+^ T cells at the single cell level. To further verify the function of all these subsets, adoptive transfers could be very useful; however, this remains challenging to set up given the many different functions to assess. Therefore, we attributed the function of the subsets to the expression of their main discerning markers. Another challenge could be the tracking of the development of memory T cell heterogeneity in time to address, e.g., the onset and waning of particular memory T cell subsets.

In this study, we have only considered cell-surface markers on T cells. However, T cell heterogeneity can be found in different levels including at the transcriptomic and epigenetic level. Moreover, intracellular proteins such as transcription factors or excreted cytokines could also provide further heterogeneity, implying that the subsets we describe differ in transcription factor profile and cytokine production. With respect to the latter, reporter mouse models for transcription factors or cytokines should help to functionally define the heterogeneous memory T cells subsets in these experiments.

### Resource availability

#### Lead contact

Further information and requests for resources and reagents should be directed to and will be fulfilled by the Lead Contact, Ramon Arens (R.Arens@lumc.nl).

#### Materials availability

Materials generated in this study will be made available on reasonable requests with a completed Materials Transfer Agreement.

#### Data and code availability

The published article includes all relevant datasets generated or analyzed during this study.

## Methods

All methods can be found in the accompanying Transparent Methods supplemental file.

## Supplemental information

Document S1. Transparent methods, figures S1–S8, and tables S1
