# Supplementary material for: Memory CD8+ T cell heterogeneity is primarily driven by pathogen-specific cues and additionally shaped by the tissue environment
Source: iScience. 2020 Dec 16;24(1):101954. doi: 10.1016/j.isci.2020.101954 (PMC7797528; doi:10.1016/j.isci.2020.101954)
Supplement: Document S1. Transparent methods, figures S1–S8, and tables S1 [file mmc1.pdf]

iScience, Volume *24*

Supplemental Information

Memory CD8$^+$ T cell heterogeneity is primarily driven by pathogen-specific cues and additionally shaped by the tissue environment

Esmé T.I. van der Gracht, Guillaume Beyrend, Tamim Abdelaal, Iris N. Pardieck, Thomas H. Wesselink, Floortje J. van Haften, Suzanne van Duikeren, Frits Koning, and Ramon Arens

Figure S1. Pathogen-specific cues induce distinct development of circulating CD8$^+$ T cells after LCMV Armstrong and LM-GP33 infection. Related to Figure 1.

(A) C57BL/6 mice were infected with LCMV Armstrong or LM-GP33. Representative plots show the gating strategy of detecting GP33-specific CD8$^+$ T cells (depicted in Figure 1B) taken from blood and analysed by flow cytometry. (B) Representative plot showing the CD44 and KLRG1 cell—surface-phenotype of GP33-specific CD8$^+$ T cells at day 45 after infection. (C) Longitudinal analysis of CD44$^+$KLRG1$^+$ GP33-specific CD8$^+$ T cells in blood. (D, E, F) tSNE maps describing the local probability density of GP33-specific CD8$^+$ T cells (D,E) and total CD8$^+$ T cells (F) stained with CD62L, CD44, and KLRG1 at day 7 (D) and day 45 (E,F) post infection.

Figure S2. CyTOF mass cytometry panel. Related to Figure 1 and Table S1.

(A) tSNE embeddings of total CD45$^+$ cells obtained from the spleen of LCMV infected mice showing the level of marker expression. (B) tSNE embeddings of total CD45$^+$ cells in the spleen from LCMV infected and control (naïve) mice showing the expression levels of APC and PE labelled tetramers. (C) tSNE embeddings of total CD8$^+$ T cells obtained from the spleen of LCMV infected mice showing the level of marker expression.

Figure S3. Gating strategy of CyTOF mass cytometry data and selection of T cell clusters. Related to Figure 1.

(A, B) Representative plots show the gating strategy of CD45$^+$ live cells (A) and GP33-specific CD8$^+$ T cells (B) obtained from the liver and analysed by mass cytometry. (C) Heatmap of liver GP33-specific CD8$^+$ T cell clusters identified at day 50 after LCMV Armstrong or LM-GP33 infection. Level of ArcSinh5transformed expression marker is displayed by a rainbow scale. (D) Average abundance of the GP33-specific CD8$^+$ T cell clusters shown in C. Data are represented as mean ± SEM. *P<0.05, Student t test. Selection of the cluster shown in Figure 1H are marked with an asteriks, and are based on an average abundance of >5%, and a significant difference between groups. (E) Principal component analysis (PCA) analysis of the liver GP33-specific CD8$^+$ T cell clusters (shown in C and D), illustrating the contribution of the different clusters to the sample distribution. The selected cluster numbers are indicated in red (cluster 7, 5 and 8).

Figure S4. Pathogen-specific cues induce distinct development of GP33-specific CD8$^+$ T cells in lungs and bone marrow. Related to Figure 2.

(A, B) Heatmaps of GP33-specific CD8$^+$ T cell clusters in lungs (A) and bone marrow (B) elicited after infection by LCMV Armstrong, MCMV-GP33 or LCMV clone 13. Clusters were selected on their abundance (>5%) and significant difference, and categorized into T_{CM}, T_{EM} and T_{RM} subsets. The level of ArcSinh5 transformed marker expression of the markers providing discernment is displayed by a rainbow scale. Bar graphs indicate the abundance and significant differences of the selected GP33-specific CD8$^+$ T cell clusters in each infection. Data are represented as mean ± SEM. *P<0.05, ANOVA.

Figure S5. Pathogen-specific cues induce distinct development of memory CD8$^+$ T cell populations. Related to Figure 2.

(A, B) Heatmaps of CD8$^+$ T cell clusters in spleen (A), liver (B), lungs (C) and bone marrow (D) elicited after infection by LCMV Armstrong, MCMV-GP33 or LCMV clone 13. Clusters were selected on their abundance (>5%) and significant difference, and categorized into T_{CM}, T_{EM} and T_{RM} subsets. The level of ArcSinh5

transformed marker expression of the markers providing discernment is displayed by a rainbow scale. Bar graphs indicate the abundance and significant differences of the selected GP33-specific $CD8^+$ T cell clusters in each infection. Data are represented as mean ± SEM. *P<0.05, ANOVA.

Figure S6. The tissue environment shapes the differentiation of memory $CD8^+$ T cell subsets after infection. Related to Figure 3.

(A-C) Heatmaps of $CD8^+$ T cell clusters elicited after infection with LCMV Armstrong (A), MCMV-GP33 (B) or LCMV clone 13 (C). Clusters were selected on their abundance (>5%) and significant difference, and categorized into T_{CM}, T_{EM} and T_{RM} subsets. The level of ArcSinh5 transformed marker expression of the markers providing discernment is displayed by a rainbow scale. Bar graphs indicate the abundance and significant differences of the selected GP33-specific $CD8^+$ T cell clusters in blood, bone marrow (BM), liver, lungs and spleen. Data are represented as mean ± SEM. *P<0.05, ANOVA.

Figure S7. Memory $CD8^+$ T cell differentiation is dominantly defined by type of infection. Related to Figure 4.

(A-D) tSNE embeddings of GP33-specific and total $CD8^+$ T cells isolated from infected mice and various organs. Cells are color coded per type of tissue or virus. (A,B) Distribution of GP33-specific $CD8^+$ T cells per tissue (A) and infection (B) in one tSNE analysis. (C, D) Distribution of total $CD8^+$ T cells per tissue (C) and infection (D) in one tSNE analysis. $CD8^+$ T cells are downsampled to 15.000 cells per sample.

Figure S8. Continuous low-level antigen triggering drives T_{EM} and T_{RM} cell differentiation. Related to Figure 5.

(A) C57BL/6 mice were infected with MCMV-FKBP-E7 or MCMV-E7. (B) Longitudinal analysis of E7-specific $CD8^+$ T cells in blood. Data are represented as mean ± SEM. Dots represent the values from individual mice. (C) Principal Component Analysis (PCA) of E7-specific $CD8^+$ T cells in spleen and liver illustrating the phenotypic distinction of these cells induced by dissimilar infection. (D, E) Heatmaps of GP33-specific $CD8^+$ T cell clusters in the spleen (D) and liver (E). Clusters were selected on their abundance (>2%) and significant difference, and categorized into T_{CM}, T_{EM} and T_{RM} subsets. The level of ArcSinh5 transformed marker expression of the markers providing discernment is displayed by a rainbow scale. Bar graphs indicate the abundance and significant differences of the selected E7-specific $CD8^+$ T cell clusters elicited by MCMV-FKBP-E7 and MCMV-E7. Data are represented as mean ± SEM. *P<0.05, Student *t* test. (F) PCA of total $CD8^+$ T cells in the spleen and liver illustrating the phenotypic distinction of the $CD8^+$ T cells induced by dissimilar infection. (G) PCA of T_{RM} and T_{EM} cell subsets in the liver illustrating the phenotypic distinction of these T cells subsets induced by dissimilar infection.

Table S1. CyTOF Mass Cytometry Panel. Anti-mouse monoclonal antibodies used for staining of cells for mass cytometry analysis. Antibodies were either purchased pre-conjugated, or antibodies were conjugated to the indicated lanthanide metal isotopes. Related to Figure 1 and Figure S2.

Antibody	Clone	Metal	Pre-conjugated	Company	Cat no	Cat no metal (Fluidigm)
**Anti-PE**	PE001	165 Ho	x	Fluidigm	3165015B	
**Anti-APC**	APC003	176 Yb	x	Fluidigm	3176007B	
**CD3e**	145-2C11	172 Yb		eBioscience	14-0031-86	201172A
**CD4**	RM4-5	145 Nd	x	Fluidigm	3145002B	
**CD8a**	53-6.7	168 Er	x	Fluidigm	3168003B	
**CD8b**	YTS156.7.7	194 Pt		BioLegend	126602	201194
**CD11a**	M17/4	160 Gd		eBioscience	16-0111-82	201160A
**CD11b**	M1/70	154 Sm	x	Fluidigm	3154006B	
**CD11c**	N418	167 Er		eBioscience	14-0114-85	201167A
**CD19**	6D5	Qdot655: 112/114 Cd	x	ThermoFisher	Q10379	
**CD25**	3C7	150 Nd	x	Fluidigm	3150002B	
**CD27**	LG.3A10	158 Gd		eBioscience	14-0272-82	201158A
**CD38**	90	163 Dy		eBioscience	14-0381-85	201163A
**CD39**	24DMS1	152 Sm		eBioscience	14-0391-82	201152A
**CD43**	1B11	115 In		BioLegend	121202	
**CD44**	IM7	142 Nd		eBioscience	14-0441-86	201142A
**CD45**	30-F11	89Y	x	Fluidigm	3089005B	
**CD49a**	Ha31/8	151 Eu		BD Biosciences	555001	201151A
**CD54**	YN1/1.7.4	164 Dy		BioLegend	116102	201164A
**CD62L**	MEL-14	169 Tm		BioLegend	104443	201169A
**CD69**	H1.2F3	143 Nd	x	Fluidigm	3143004B	
**CD73**	TY/23	148 Nd		BD Biosciences	550738	201148A
**CD86**	GL1	171 Yb		eBioscience	14-0862-85	201171A
**CD103**	2.E7	173 Yb		eBioscience	14-1031-85	201173A
**CD122**	TM-b1	155 Gd		eBioscience	14-1222-85	201155A
**CD127**	A7R34	175 Lu	x	Fluidigm	3175006B	
**CD160**	7H1	209 Bi		BioLegend	143002	
**CD161**	PK136	170 Er	x	Fluidigm	3170002B	
**CD223**	eBioC9B7W	161 Dy		eBioscience	14-2231-85	201161A
**CD278**	7E.17G9	162 Dy		eBioscience	14-9942-85	201162A
**CX3CR1**	SA011F11	174 Yb		BioLegend	149002	201174A
**CXCR3**	CXCR3-173	149 Sm		eBioscience	16-1831-85	201149A
**CXCR5**	L138D7	153 Eu		BioLegend	145502	201153A
**CXCR6**	SA051D1	144 Nd		BioLegend	151102	201144A
**FR4**	TH6	198 Pt		BioLegend	125102	201198
**KLRG-1**	2F1	166 Er		eBioscience	16-5893-85	201166A
**Ly6C**	HK1.4	156 Gd		eBioscience	16-5932-85	201156A
**NKG2A**	20d5	147 Sm		eBioscience	16-5896-85	201147A
**PD-1**	29F.1A12	159 Tb	x	Fluidigm	3159024B	
**Sca-1**	D7	141 Pr		BioLegend	108135	201141A
**TCRgd**	eBioGL3	146 Nd		eBioscience	14-5711-85	201146A

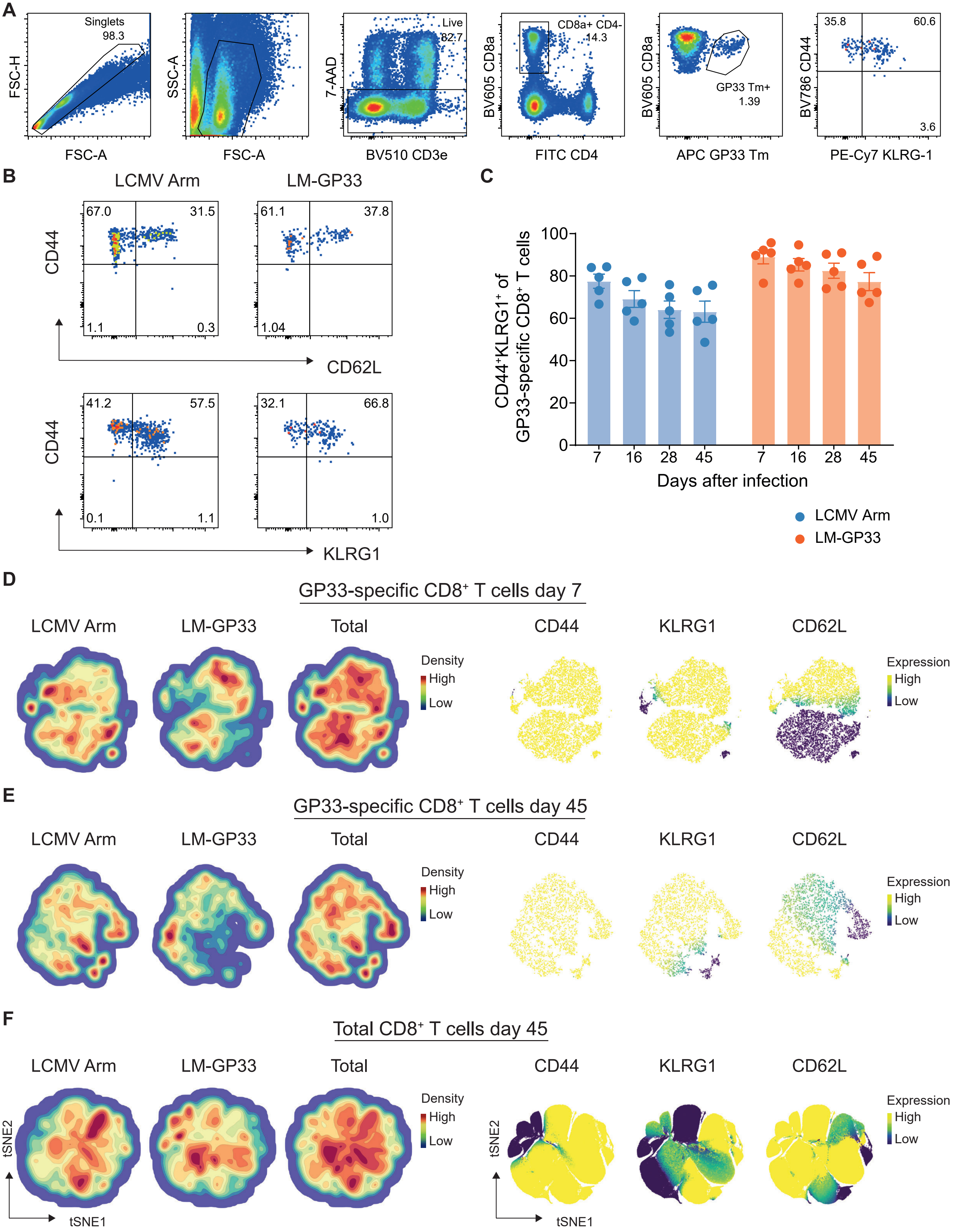

Figure S1. Pathogen-specific cues induce distinct development of circulating CD8⁺ T cells after LCMV Armstrong and LM-GP33 infection. Related to Figure 1.

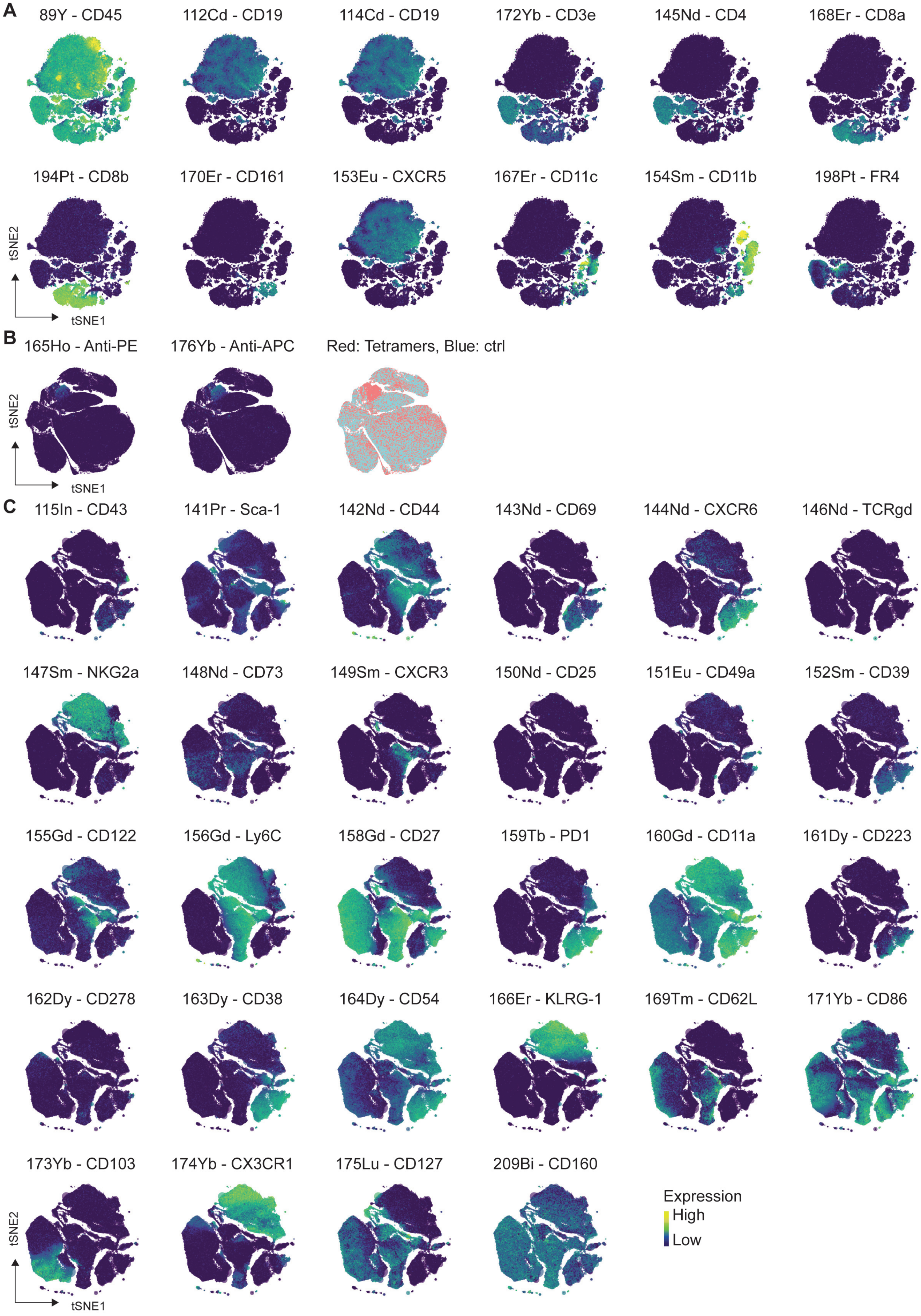

Figure S2. CyTOF mass cytometry panel. Related to Figure 1 and Table S1.

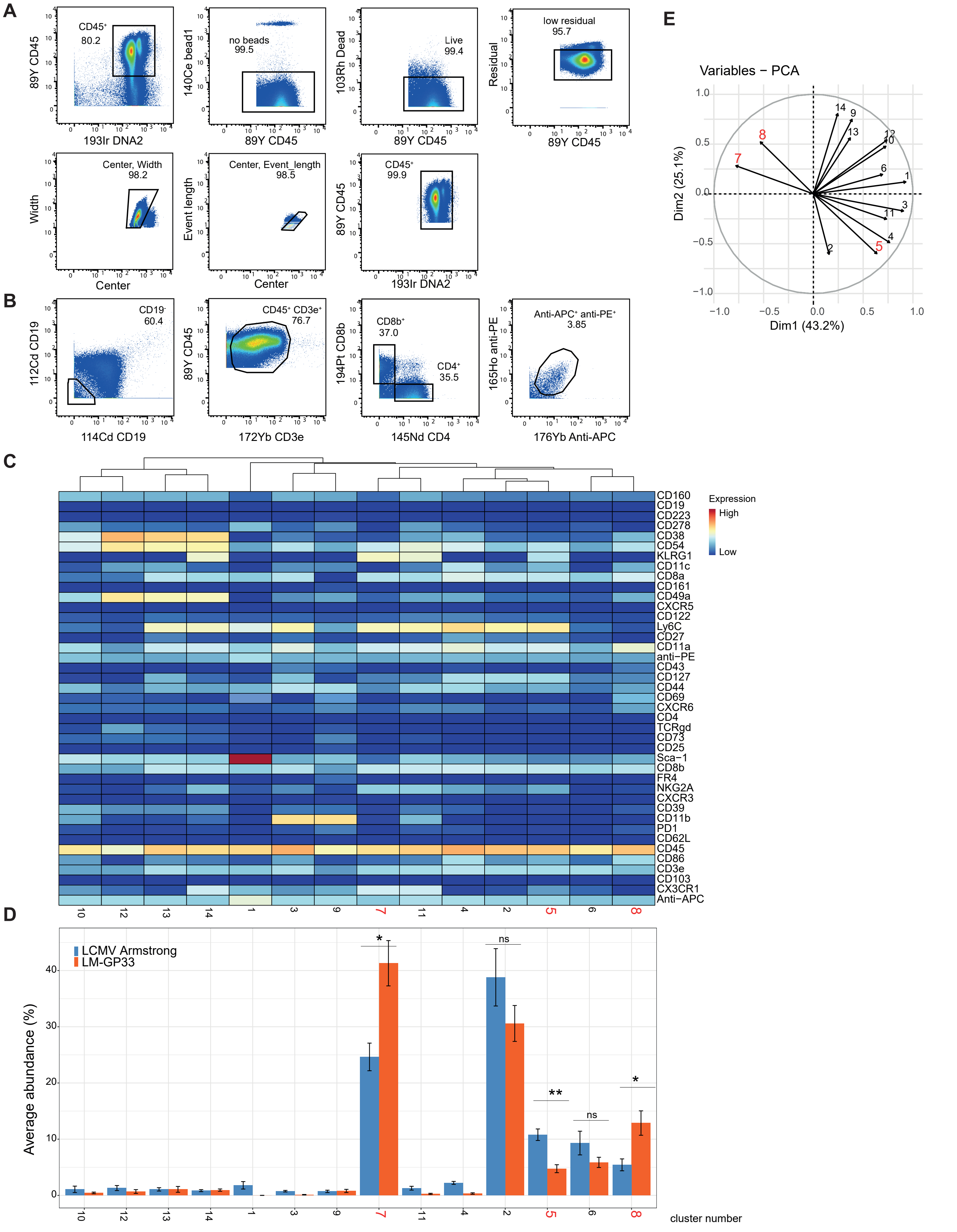

Figure S3. Gating strategy of CyTOF mass cytometry data and selection of T cell clusters. Related to Figure 1.

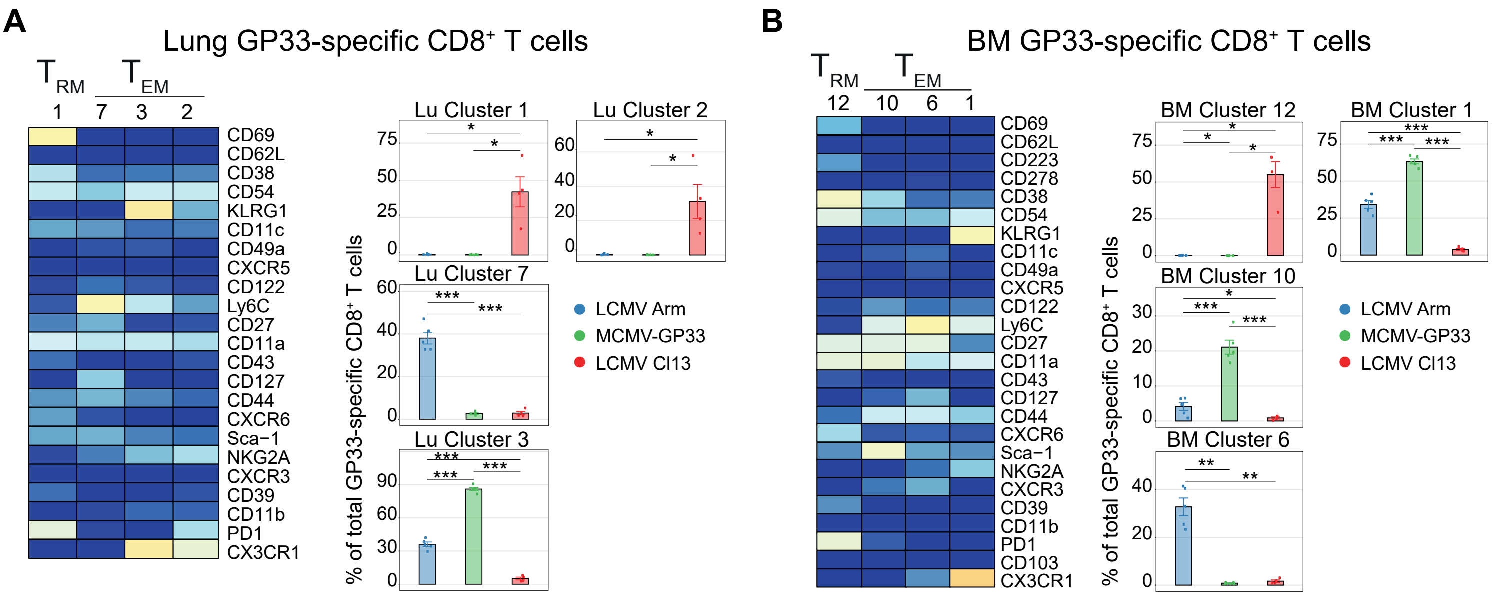

Figure S4. Pathogen-specific cues induce distinct development of GP33-specific CD8+ T cells in lungs and bone marrow. Related to Figure 2.

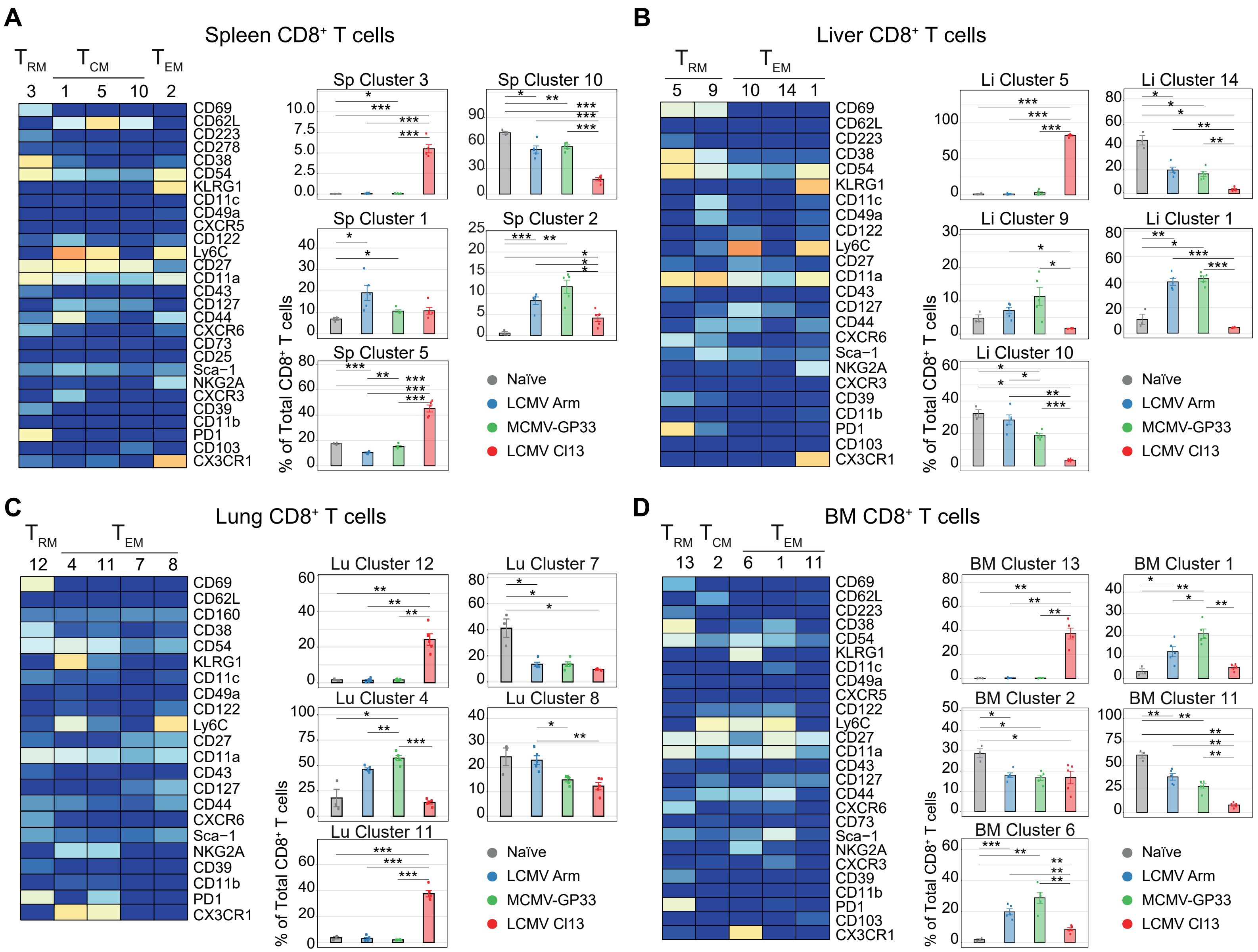

Figure S5. Pathogen-specific cues induce distinct development of memory CD8$^+$ T cell populations. Related to Figure 2.

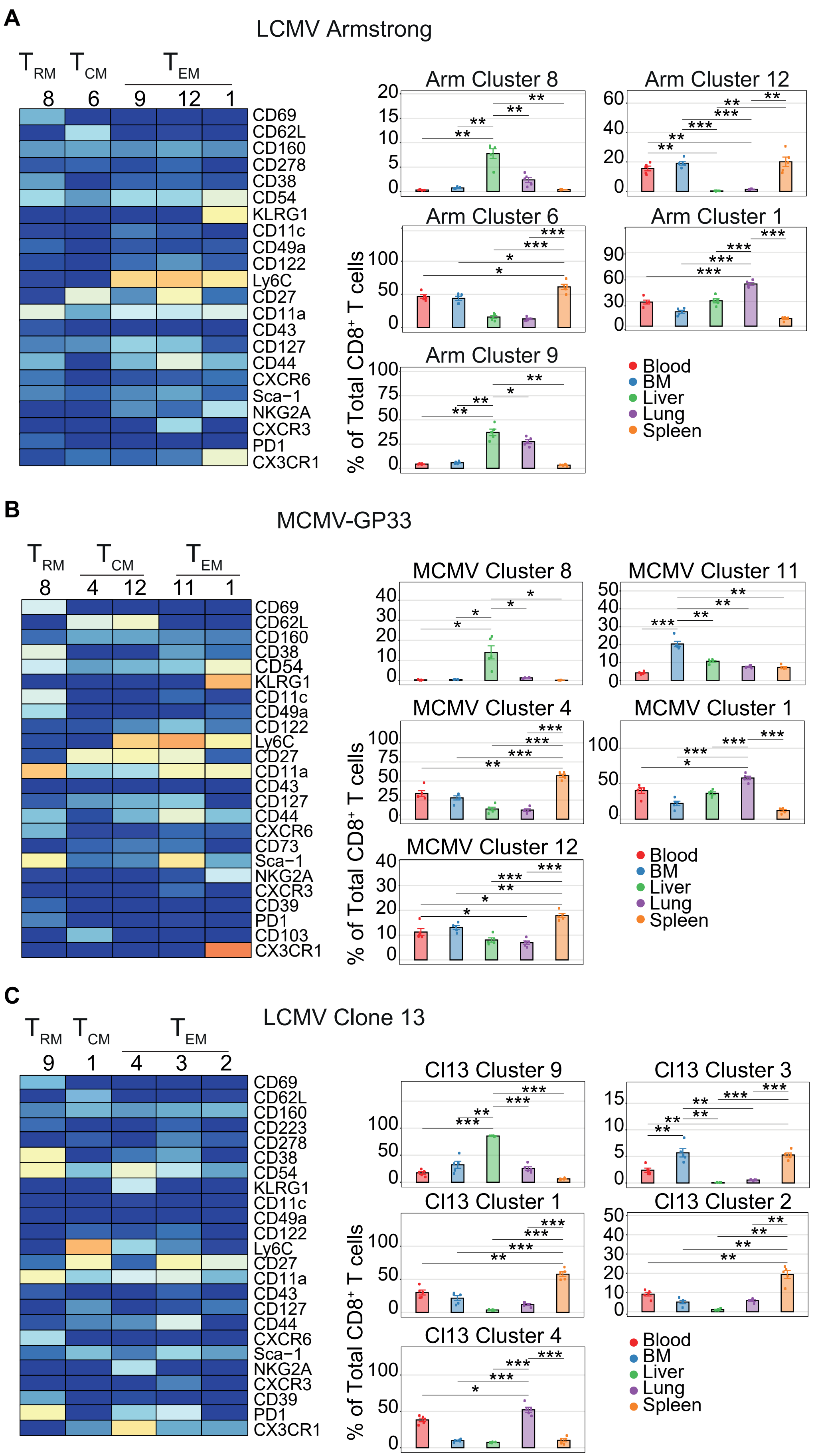

Figure S6. The tissue environment shapes the differentiation of memory $CD8^{+}$ T cell subsets after infection. Related to Figure 3.

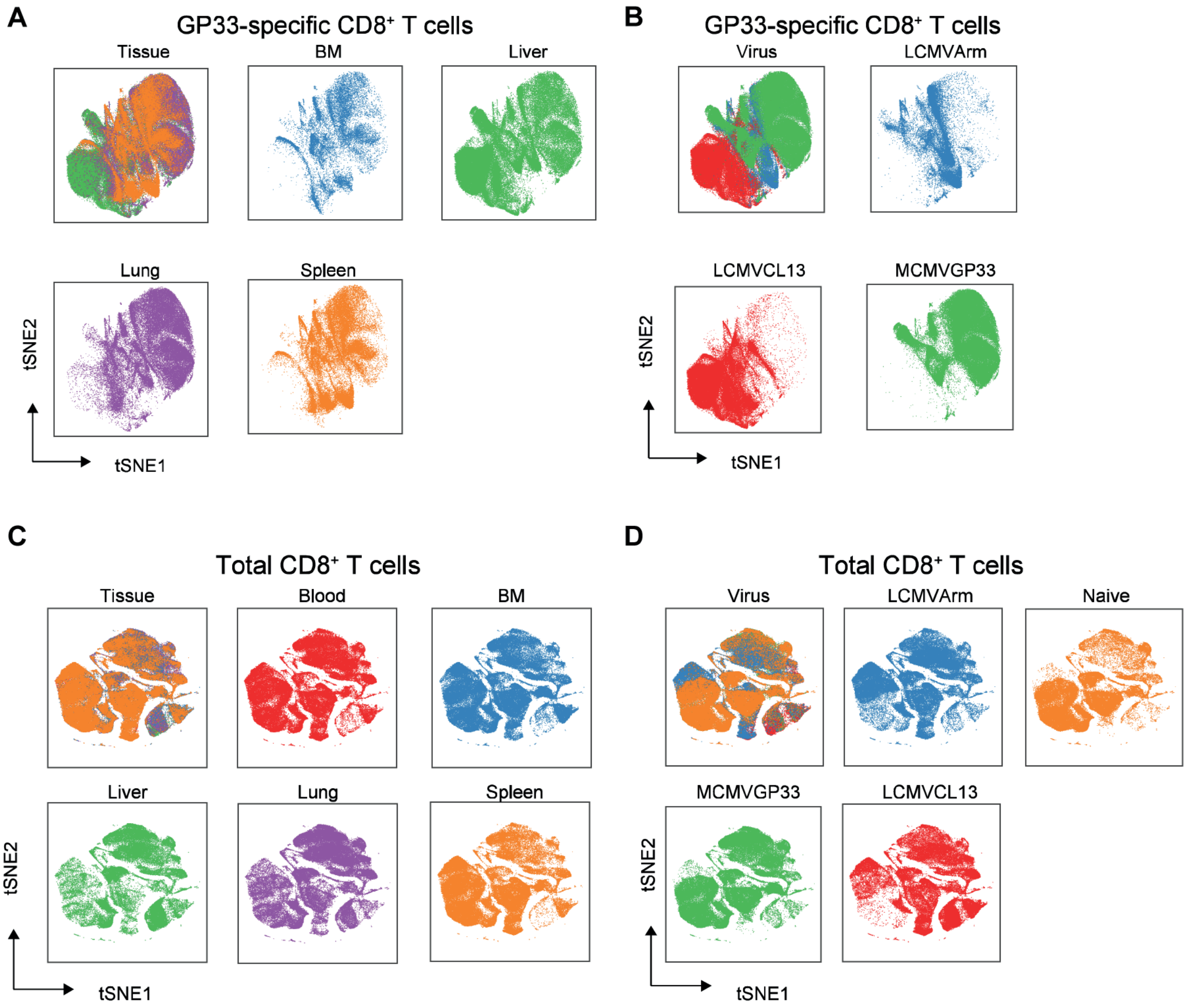

Figure S7. Memory CD8$^+$ T cell differentiation is dominantly defined by type of infection. Related to Figure 4.

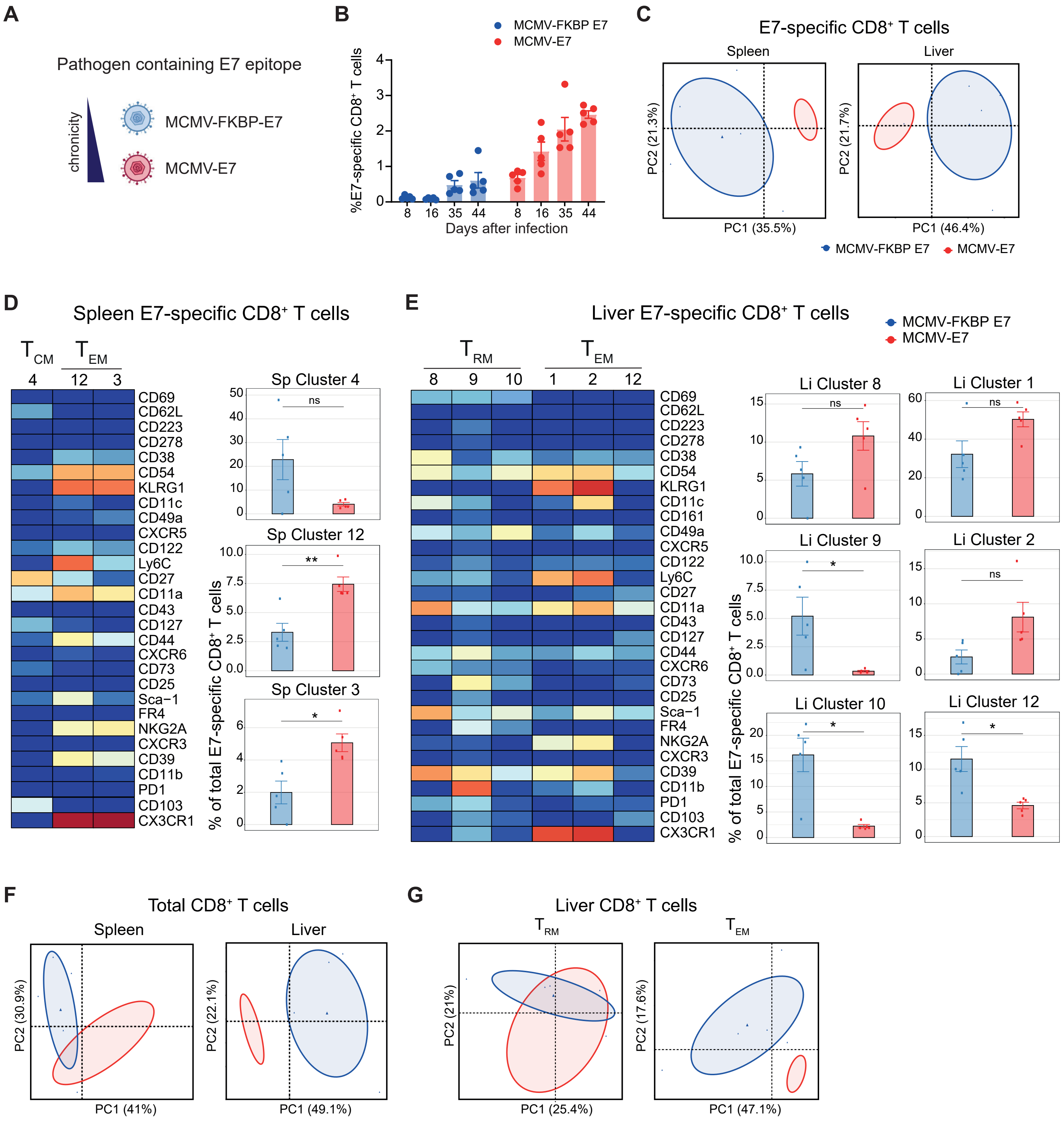

Figure S8. Continuous low-level antigen triggering drives T_{EM} and T_{RM} cell differentiation. Related to Figure 5.

Transparant Methods

Mice

C57BL/6 mice were obtained from Charles River Laboratories (L'Arbresle, France) or Jackson Laboratory (Sacramento, CA, USA). At the start of the experiments, mice were six to eight-weeks-old. Animals were housed in individually ventilated cages under specific-pathogen free conditions at the animal facility at Leiden University Medical Center (LUMC). All animal experiments were approved by the Animal Experiments Committee of the LUMC and performed according to the recommendations and guidelines set by LUMC and by the Dutch Experiments on Animals Act.

Viral and bacterial infections

Mice were infected with lymphocytic choriomeningitis virus (LCMV) Armstrong (2×10^5 PFU, intraperitoneally), LCMV Clone 13 (2×10^6 plaque-forming units (PFU), intravenously via retro-orbital injection), *Listeria monocytogenes* (LM) expressing GP33 (LM-GP33 ((Zenewicz et al., 2002); 1×10^4 colony-forming units (CFU), intravenously via retro-orbital injection), MCMV-GP33 (2×10^5 PFU, intraperitoneally), MCMV-E7 (1×10^6 PFU, intraperitoneally) or MCMV-FKBP-E7 (1×10^6 PFU, intraperitoneally). For co-infection studies, mice were infected intraperitoneally with 1×10^5 PFU MCMV-GP33, 1×10^5 PFU LCMV Armstrong or a combination of MCMV-GP33 and LCMV Armstrong (each 1×10^5 PFU).

LCMV Armstrong and LCMV clone 13 were propagated in BHK cells, and titers were determined by plaque assays on Vero cells as described previously (Ahmed et al., 1984). MCMV-GP33, MCMV-E7, and the single-cycle replicating MCMV-FKBP-E7 were generated and reconstituted as described elsewhere (Beyranvand Nejad et al., 2019; Welten et al., 2015). In brief, nucleotide sequences encoding the $GP_{33\text{-}41}$ epitope (GP33; KAVYNFATC) of LCMV or the $E7_{49\text{-}57}$ epitope (E7; RAHYNIVTF) of human papilloma virus were inserted by targeted mutagenesis at the C-terminus of the IE2 genes.

Isolation of lymphocytes

Peripheral blood was collected from the tail vein. Splenocytes were obtained by mincing the tissue through cell strainers. Bone marrow cells were obtained from the femurs and tibias by centrifugation. Blood cells, splenocytes and bone marrow cells were depleted of erythrocytes using ammonium chloride buffer. Subsequently, T cells were isolated using MicroBeads (130-095-130, Miltenyi Biotec).

To remove remaining circulating blood cells from the liver and lungs, mice were perfused with 20 ml PBS containing 2 mM EDTA. Next, liver and lungs were cut into small pieces using surgical knives. Liver tissue

was resuspended in 3.5 ml IMDM containing 250 U/ml collagenase type 1-A (C2674, Sigma) and 20 µg/ml DNase I (D5025, Sigma), and lung tissue was incubated with 1 ml IMDM and 250 U/ml collagenase and 20 µg/ml DNase. After incubation with collagenase/DNase for 25 minutes at 37°C, the liver and lung tissue were dissociated into single-cell suspensions using 70 µm cell strainers, and subsequently lymphocytes were isolated using a Percoll (GE Healthcare) gradient.

Conjugation of antibodies for CyTOF analysis

Metal-conjugated antibodies were either purchased from Fluidigm or were generated by conjugation of lanthanide metal isotopes to anti-mouse antibodies using the Maxpar X8 Polymer method according to the manufacturer's protocol (Fluidigm). Cisplatins 194 and 198 were conjugated to anti-mouse monoclonal antibodies using a modified protocol as previously described (Mei et al., 2016). Conjugation with 209 Bismuth was performed using a protocol adapted from M. Spitzer (Spitzer et al., 2017). All in-house conjugated antibodies were diluted to 0.5 mg/ml in antibody stabilizer supplemented with 0.05% sodium azide (Candor Biosciences). Serial dilution staining was performed on mouse lymphocytes to determine appropriate antibody dilution.

Flow cytometry

Fluorescently-labelled monoclonal anti-mouse antibodies against the following cell-surface molecules were used: CD3 (clone 145-2C11, BD Biosciences), CD4 (clone RM4-5, BioLegend), CD8 (clone 53-6.7, BioLegend), CD127 (clone A7R34, ThermoFisher), KLRG1 (clone 2F1, ThermoFisher), CD44 (clone IM7, BioLegend), CD62L (clone MEL-14, BioLegend), CD69 (clone H1.2F3, BD Biosciences) and CD38 (clone 90, Thermo Fisher). Cells were stained according to our previously published protocol (Arens et al., 2011). 7-AAD (A1310, Invitrogen) cell viability dye was used to exclude dead cells. GP33-specific $CD8^+$ T cells were detected using MHC class I tetramers for the KAVYNFATC epitope, and E7-specific $CD8^+$ T cells were detected using MHC class I tetramers for the RAHYNIVTF epitope. Flow cytometric acquisition was performed on a BD Fortessa flow cytometer (BD Biosciences).

CyTOF mass cytometry

Approximately 3×10^6 cells per sample were stained for mass cytometry analysis. First, cells were stained with PE and APC-labelled MHC class I tetramers for 30 minutes on ice in PBS containing 0.5% FCS. Cells were washed and subsequently incubated for 20 minutes with 1 µM Interchalator-Rh (201103A, Fluidigm) in Maxpar Cell Staining buffer (201068, Fluidigm) on ice. Subsequently, a-specific binding was prevented

by incubating cells with Fc block (clone 2.4G2, anti-CD16/anti-CD32 antibody) and mouse serum for 15 minutes. Metal conjugated anti-PE and anti-APC antibodies were added in a final dilution of 1:50 and incubated for 45 minutes. The antibody mixture containing all other metal-conjugated antibodies was added and incubated for an additional 45 minutes. After washing, the cells were incubated overnight with 25 nM Intercalator-Ir (201192A, Fluidigm) in Maxpar Fix and Perm Buffer (201067, Fluidigm). Cells were pelleted in staining buffer and measured within one week. Before measuring, EQ™ Four Element Calibration Beads (201078, Fluidigm) were added in a 1:10 ratio to normalize the short-term signal fluctuations during the course of each experiment (Finck et al., 2013). Samples were measured on a CyTOF Helios mass cytometer.

Flow cytometry data analysis

Flow cytometry data was analysed using FlowJo or Cytosplore. For Cytosplore analysis, samples were analysed by hierarchical stochastic neighbourhood embedding (HSNE) (van Unen et al., 2017) based on approximated t-distributed stochastic neighbourhood embedding (A-tSNE) (Pezzotti et al., 2017). Statistical analyses were performed using GraphPad Prism (La Jolla, CA, Unites States). The Mann-Whitney or Student *t* test was used for statistical analysis. All P values were two-sided, and $P < 0.05$ was considered statistically significant.

Mass cytometry data analysis

We set our gating strategy to live single cells, positive for CD45, and excluded reference beads. For further analysis, live $CD45^+$ gated files were compensated using Catalyst (Chevrier et al., 2018). Total $CD8^+$ T cells and MHC class I tetramer-specific $CD8^+$ T cells were selected in FlowJo for subsequent analysis. Marker expression was ArcSinh5 transformed and subjected to dimensionality reduction analyses in Cytosplore (Höllt et al., 2016) or FlowSOM (Van Gassen et al., 2015). For Cytosplore analysis, samples were analysed by hierarchical stochastic neighbourhood embedding (HSNE) (van Unen et al., 2017) based on approximated t-distributed stochastic neighbourhood embedding (A-tSNE) (Pezzotti et al., 2017).

FlowSOM was used for the identification of tissue and virus-specific clusters. Using FlowSOM, 14 clusters were identified per analysis. Subsequently, *Cytofast* (Beyrend et al., 2018; Beyrend et al., 2019) was used for visualization and quantification of cell clusters as well as principal component analyses (PCA). To reveal the association of clusters with certain groups, clusters were selected based on the size of the cluster (abundance of at least >5% of total are shown) and significance. PCA of T_{RM} and T_{EM} $CD8^+$ T cell subsets as shown in figure 5F and figure S6G were selected in Cytosplore by expression of CD69 and CD62L and further analysed using *Cytofast*.

T_{RM} (CD62L$^-$ CD69$^+$), T_{CM} (CD62L$^+$ CD69$^-$) and T_{EM} (CD62L$^-$ CD69$^-$) CD8$^+$ T cell clusters were selected by expression of CD69 and CD62L. For visualization of the memory T cell subsets adjusted scaling of CD69 and CD62L was used. For visualization we excluded markers that were used to gate the GP33-specific memory T cells (represented by the inclusion markers CD45, CD8a, CD8b, MHC class I tetramers, and the exclusion markers CD19, TCRgd, CD4), and markers that were not providing any discernment (for example due to lack of expression on the CD8$^+$ memory T cells).

The similarity between tSNE maps was quantified using the Jensen-Shannon (JS) divergence. The tSNE maps were first converted into two-dimensional probability density functions. Next, the JS divergence was used to measure the similarity between the two maps. Using a base 2 log, the JS divergence values ranged from 0 (indicating identical distributions) to 1 (indicating disjoint distributions). The dual tSNE analysis was performed to quantify the individual samples similarity based on the clusters composition. The dual tSNE analysis was performed as described by van Unen *et al.* (van Unen et al., 2016). Briefly, a data matrix ($n_{samples}$ x $m_{clusters}$) containing the cluster frequencies of the individual samples was used as input to generate the samples tSNE map, hence samples with similar profiles across clusters end up close together in the map. The data matrix was normalized by centering (zero mean) and scaling (unit variance). The clusters tSNE map was obtained by using the transposed normalized data matrix as input, hence clusters with similar profiles across individual samples end up close together in the map.

The Student *t* test or ANOVA was used for statistical analysis. All P values were two-sided, and $P < 0.05$ was considered statistically significant.

References

Ahmed, R., Salmi, A., Butler, L.D., Chiller, J.M., and Oldstone, M.B. (1984). Selection of genetic variants of lymphocytic choriomeningitis virus in spleens of persistently infected mice. Role in suppression of cytotoxic T lymphocyte response and viral persistence. J Exp Med *160*, 521-540.

Arens, R., Loewendorf, A., Redeker, A., Sierro, S., Boon, L., Klenerman, P., Benedict, C.A., and Schoenberger, S.P. (2011). Differential B7-CD28 costimulatory requirements for stable and inflationary mouse cytomegalovirus-specific memory CD8 T cell populations. J Immunol *186*, 3874-3881.

Beyranvand Nejad, E., Ratts, R.B., Panagioti, E., Meyer, C., Oduro, J.D., Cicin-Sain, L., Fruh, K., van der Burg, S.H., and Arens, R. (2019). Demarcated thresholds of tumor-specific CD8 T cells elicited by MCMV-based vaccine vectors provide robust correlates of protection. J Immunother Cancer *7*, 25.

Beyrend, G., Stam, K., Hollt, T., Ossendorp, F., and Arens, R. (2018). Cytofast: A workflow for visual and quantitative analysis of flow and mass cytometry data to discover immune signatures and correlations. Comput Struct Biotechnol J *16*, 435-442.

Beyrend, G., Stam, K., Ossendorp, F., and Arens, R. (2019). Visualization and Quantification of High-Dimensional Cytometry Data using Cytofast and the Upstream Clustering Methods FlowSOM and Cytosplore. J Vis Exp.

Chevrier, S., Crowell, H.L., Zanotelli, V.R.T., Engler, S., Robinson, M.D., and Bodenmiller, B. (2018). Compensation of Signal Spillover in Suspension and Imaging Mass Cytometry. Cell Systems *6*, 612-620.e615.

Finck, R., Simonds, E.F., Jager, A., Krishnaswamy, S., Sachs, K., Fantl, W., Pe'er, D., Nolan, G.P., and Bendall, S.C. (2013). Normalization of mass cytometry data with bead standards. Cytometry A *83*, 483-494.

Höllt, T., Pezzotti, N., van Unen, V., Koning, F., Eisemann, E., Lelieveldt, B., and Vilanova, A. (2016). Cytosplore: Interactive Immune Cell Phenotyping for Large Single-Cell Datasets. Computer Graphics Forum *35*, 171-180.

Mei, H.E., Leipold, M.D., and Maecker, H.T. (2016). Platinum-conjugated antibodies for application in mass cytometry. Cytometry Part A *89*, 292-300.

Pezzotti, N., Lelieveldt, B.P.F., Van Der Maaten, L., Hollt, T., Eisemann, E., and Vilanova, A. (2017). Approximated and User Steerable tSNE for Progressive Visual Analytics. IEEE Trans Vis Comput Graph *23*, 1739-1752.

Spitzer, M.H., Carmi, Y., Reticker-Flynn, N.E., Kwek, S.S., Madhireddy, D., Martins, M.M., Gherardini, P.F., Prestwood, T.R., Chabon, J., Bendall, S.C.*, et al.* (2017). Systemic Immunity Is Required for Effective Cancer Immunotherapy. Cell *168*, 487-502.e415.

Van Gassen, S., Callebaut, B., Van Helden, M.J., Lambrecht, B.N., Demeester, P., Dhaene, T., and Saeys, Y. (2015). FlowSOM: Using self-organizing maps for visualization and interpretation of cytometry data. Cytometry A *87*, 636-645.

van Unen, V., Höllt, T., Pezzotti, N., Li, N., Reinders, M.J.T., Eisemann, E., Koning, F., Vilanova, A., and Lelieveldt, B.P.F. (2017). Visual analysis of mass cytometry data by hierarchical stochastic neighbour embedding reveals rare cell types. Nature Communications *8*, 1740.

van Unen, V., Li, N., Molendijk, I., Temurhan, M., Hollt, T., van der Meulen-de Jong, A.E., Verspaget, H.W., Mearin, M.L., Mulder, C.J., van Bergen, J.*, et al.* (2016). Mass Cytometry of the Human Mucosal Immune System Identifies Tissue- and Disease-Associated Immune Subsets. Immunity *44*, 1227-1239.

Welten, S.P., Redeker, A., Franken, K.L., Oduro, J.D., Ossendorp, F., Cicin-Sain, L., Melief, C.J., Aichele, P., and Arens, R. (2015). The viral context instructs the redundancy of costimulatory pathways in driving CD8(+) T cell expansion. Elife *4*, e07486.

Zenewicz, L.A., Foulds, K.E., Jiang, J., Fan, X., and Shen, H. (2002). Nonsecreted Bacterial Proteins Induce Recall CD8 T Cell Responses But Do Not Serve as Protective Antigens. J Immunol *169*, 5805-5812.
